# Putative Genes and Pathways Involved in the Acne Treatment of Isotretinoin via Microarray Data Analyses

**DOI:** 10.1155/2020/5842795

**Published:** 2020-06-29

**Authors:** Biao Chen, Peishan Li, Jun Li, Jinping Chen

**Affiliations:** Department of Dermatology, Guangzhou Women and Children's Medical Center, Guangzhou Medical University, Guangzhou 510623, China

## Abstract

Acne is the eighth most common disease worldwide. Disease burden of acne such as anxiety, reduced self-esteem, and facial scarring lowers the life quality of acne patients. Isotretinoin is the most potent treatment for moderate-severe acne. However, the adverse events of isotretinoin especially teratogenicity limit its use. This study aims at investigating the therapeutical mechanisms of isotretinoin using bioinformatics analysis. Differentially expressed genes (DEGs) were filtered from microarray datasets GSE10432, GSE10433, and GSE11792. Functional and pathway enrichment analyses of DEGs were performed. Protein–protein interaction (PPI) network and module analyses were also conducted based on DEGs. Using isotretinoin for 1 week, *LCN2*, *PTGES*, and *GDF15* were upregulated and might mediate sebocytes apoptosis and thus decreased sebum production; *CCL2* originated from activated TNF signaling pathway and *S100A7* could be related with “acne-flare”. While treating with isotretinoin for 8 weeks, key genes were downregulated, including *HMGCS1*, *HMGCR*, *FDFT1*, *MVD*, *IDI1*, and *FDPS*, which may be associated with decreased sebum synthesis; *HMGCS1*, *HMGCR*, and *FDFT1* also probably associated with apoptosis of sebocytes. There were only two common genes including *ACSBG1* and *BCAT2* which worked in both 1 week and 8 weeks and could associate with decreased sebum synthesis and apoptosis of sebocytes, respectively. These results indicate potential therapeutics and side effect mechanisms of isotretinoin in the acne treatment and provide a research direction to further investigate the therapeutic mechanism of isotretinoin and thus develop retinoid-like compounds with similar curative effect and without teratogenicity.

## 1. Introduction

Acne is the eighth most common disease worldwide with a morbidity of 94% according to the Global Burden of Disease Project [1]. Disease burden of acne includes anger, depression, anxiety, reduced self-esteem, facial scarring, and impaired social interaction, which usually negatively impact the quality of life of acne patients [2]. Treatments of acne include topical medication such as antibiotics, benzoyl peroxide, and retinoids; and systemic medication such as isotretinoin, tetracyclines, and spironolactone [3]; as well as other miscellaneous therapies including chemical peels, intralesional injection of triamcinolone acetonide, and laser and light therapies [4]. Among them, isotretinoin is the first line and the most potent treatment for moderate-severe acne [4, 5]. The adverse events of isotretinoin include elevated liver enzymes [6] and serum lipids, xerosis, cheilitis, eye dryness, irritation and conjunctival injection, fatigue, depressed mood, arthralgia, and teratogenicity [7]. Among them, teratogenicity is the main and disastrous side effect of isotretinoin. Considering the therapeutic effect and adverse events, there is an emerging need to explore the mechanism of acne treatment with isotretinoin, and thus develop retinoid-like compounds with similar curative effect and without teratogenicity.

Microarray is a high-throughput technology which exhibits the overall gene expression data of recruited specimens from different diseases. NCBI-Gene Expression Omnibus database (NCBI-GEO) (https://www.ncbi.nlm.nih.gov/geo) is a free public website and archives for functional genomics datasets, in which microarray data are usually deposited and available. Nelson et al. performed microarray analyses of skin biopsies from acne patients treated with isotretinoin for 1 week and cultured human sebaceous gland cells (SEB-1 sebocytes) incubated with isotretinoin for 72 hours and found that *lipocalin 2* is the most highly upregulated gene induced by isotretinoin, which mediates the apoptosis of sebaceous glands of acne patients [8]. Nelson et al. also conducted microarray analyses of skin biopsies from acne patients treated with isotretinoin for 8 weeks and found that isotretinoin significantly reduces the size of the sebaceous gland at 8 weeks with a trend observed at 1 week, and only 3 common DEGs were found in samples of acne patients treated with isotretinoin at 8 weeks and 1 week, indicating that isotretinoin induces temporal changes in gene expression of acne patients' skin [9]. Bioinformatical analyses of multiple microarray datasets derived from different studies contribute to identify the hub genes and pathways which participate in the acne treatment of isotretinoin, thus provide potential therapeutic targets of acne.

In the present study, three microarray data including GSE10432, GSE10433, and GSE11792 were obtained from NCBI-GEO. Common DEGs between GSE10432 and GSE10433, DEGs in GSE11792 were filtered via the online tool GEO2R. Gene Ontology (GO) and Kyoto Encyclopedia of Genes and Genomes (KEGG) pathway enrichment analyses of DEGs were conducted using the Database for Annotation, Visualization, and Integrated Discovery (DAVID) (https://david.ncifcrf.gov/). PPI networks of DEGs were constructed via the free public online website STRING (https://string-db.org/, version 11) and free software Cytoscape; in which the hub genes and modules were further assessed to demonstrate the functions of the DEGs. This study aimed at obtaining deep insights into the therapeutic mechanism of acne treatment using isotretinoin.

## 2. Materials and Methods

### 2.1. Microarray Data and Identification of DEGs

GSE10432, GSE10433, and GSE11792 were downloaded from NCBI-GEO to identify the DEGs between SEB-1 treated with vehicle control and isotretinoin or between acne patients before and after treatment with isotretinoin. The microarray profile GSE10432 was based on the GPL8300 platform (Affymetrix Human Genome U95 Version 2 Array, Palo Alto, CA, USA) and comprised 3 biological replicates of SEB-1 treated with isotretinoin or vehicle control (Submission date: Feb 07, 2008) [8]. GSE10433 was based on the GPL571 platform (Affymetrix Human Genome U133A 2.0 Array, Palo Alto, CA, USA) and included 6 acne patients at baseline and treated with isotretinoin for 1 week (Submission date: Feb 07, 2008) [8]. GSE11792 was based on the GPL571 platform (Affymetrix Human Genome U133A 2.0 Array, Palo Alto, CA, USA) and consisted of 8 acne patients at baseline and treated with isotretinoin for 8 weeks (Submission date: Jun 16, 2008) [9].

The DEGs in GSE10432, GSE10433, and GSE11792 were analyzed using the online tool GEO2R (https://www.ncbi.nlm.nih.gov/geo/geo2r/). The adjusted *P* value and ∣logFC∣ were calculated; and *P* < 0.05 and ∣logFC | ≥0.263034405833794, 0.263034405833794, and 0.584962501 were used as a cut-point for the statistically significant DEGs in GSE10432, GSE10433, and GSE11792, respectively. The Benjamini and Hochberg false discovery rate method was used as a correction factor for the adjusted *P* value in GEO2R.

### 2.2. GO and Pathway Enrichment Analyses of DEGs in Acne Treated with Isotretinoin

GO analyses, consisting of molecular function (MF), cellular component (CC), and biological process (BP), were used to elucidate gene functions. DAVID is a free online website available for analyzing the biological meaning behind a large list of genes. In our study, GO and KEGG pathway enrichment analyses of significant DEGs, including the common DEGs between GSE10432 and GSE10433 and the DEGs in GSE11792, were conducted by DAVID, with a threshold of *P* value < 0.05 and enrichment gene count > 2.

### 2.3. Establishment of PPI Network and Modular Analysis

The common DEGs between GSE10432 and GSE10433 and DEGs in GSE11792 were analyzed using the online tool STRING, with 0.400 (medium confidence) and 0.700 (high confidence) as the minimum required interaction score, respectively. The PPI network of the above DEGs was established using Cytoscape, respectively. The node degree of the PPI network was calculated by Network Analyzer in Cytoscape. The hub genes of these PPI networks were filtered by CytoHubba, which is one of the Apps in Cytoscape. Modular analysis of the PPI network of GSE11792 was conducted using MCODE, with a Degree Cutoff = 2, Node Score Cutoff = 0.2, K − Core = 2, and MaxDepth = 100. Finally, KEGG pathway enrichment analyses of these candidate genes in every cluster of PPI networks of GSE11792 were conducted by DAVID.

## 3. Results

### 3.1. The Volcano Plots of Genes Expression and the Common DEGs of GSE10432, GSE10433, and GSE11792

**Figure 1:**
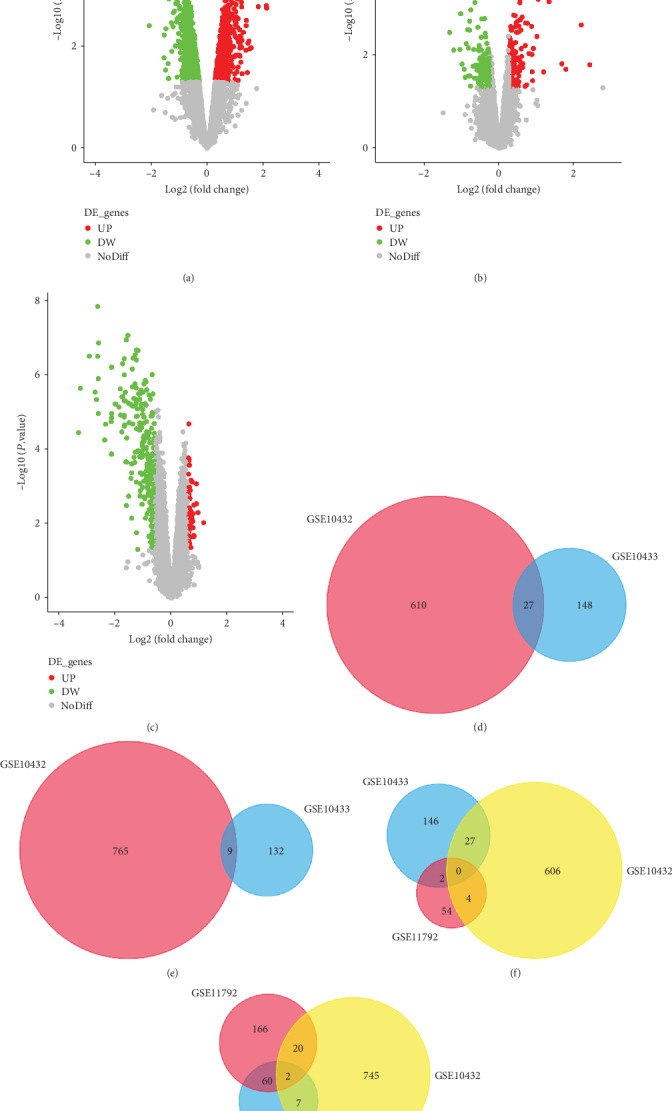
Volcano plots of genes expression and the common DEGs in GSE10432, GSE10433, and GSE11792. Volcano plots of genes expression in microarray data GSE10432 (a), GSE10433 (b), and GSE11792 (c). In volcano plots, green represents downregulated genes, red represents upregulated genes, and gray means no significant DEGs. UP: upregulated DEGs; DW: downregulated DEGs; NoDiff: nondifferentially expressed genes. (d) The common upregulated DEGs of GSE10432 and GSE10433. (e) The common downregulated DEGs of GSE10432 and GSE10433. (f) The common upregulated DEGs among GSE10432, GSE10433, and GSE11792. (g) The common downregulated DEGs among GSE10432, GSE10433, and GSE11792. Different colors indicate different datasets. The cross part represents the common DEGs.

**Figure 2:**
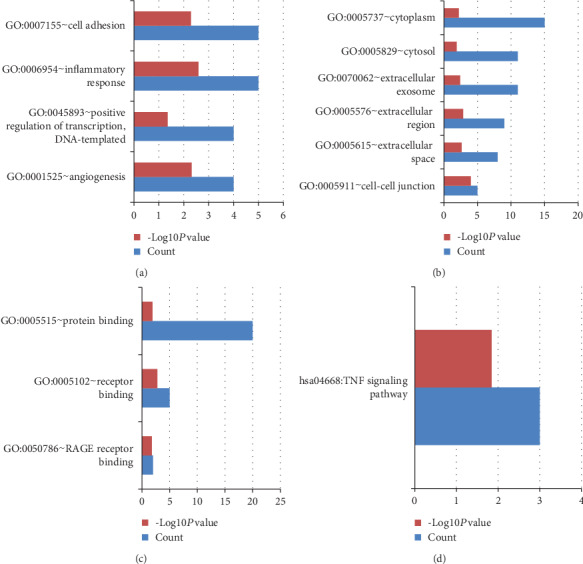
GO and KEGG pathway enrichment analyses of the upregulated DEGs in GSE10432 and GSE10433. (a), (b), (c), and (d) represent BP, CC, MF, and KEGG, respectively. Count: number of DEGs.

Three microarray datasets including GSE10432, GSE10433, and GSE11792 were downloaded from GEO. Basing on a threshold of *P* value < 0.05 and ∣log fold change (FC)  | ≥0.263034405833794, namely, ∣FC | ≥1.2, 638 upregulated and 775 downregulated genes in GSE10432, and 176 upregulated and 142 downregulated genes in GSE10433 were obtained. There were 62 upregulated and 249 downregulated genes in GSE11792 with a cut off value of *P* value < 0.05 and ∣log FC | ≥0.584962501, namely, ∣FC | ≥1.5. The volcano plots of gene expression of GSE10432 (Figure 1(a)), GSE10433 (Figure 1(b)), and GSE11792 (Figure 1(c)) were generated using a free online website imageGP (http://www.ehbio.com/ImageGP/index.php/Home/Index/Volcanoplot.html). There were 27 upregulated (Figure 1(d)) and 9 downregulated (Figure 1(e)) genes in GSE10432 and GSE10433 concurrently (Table S1). 62 upregulated genes and 249 downregulated genes were observed in GSE11792 (Table S2). There were consistently 0 upregulated (Figure 1(f)) and 2 downregulated (Figure 1(g)) genes, including *ACSBG1* and *BCAT2*, among GSE10432 (SEB-1 sebocytes incubated with isotretinoin for 72 hours), GSE10433 (acne patients treated with isotretinoin for 1 week), and GSE11792 (acne patients treated with isotretinoin for 8 weeks).

### 3.2. GO, KEGG Pathway Enrichment Analyses and PPI Network of the Common DEGs in GSE10432 and GSE10433

**Figure 3:**
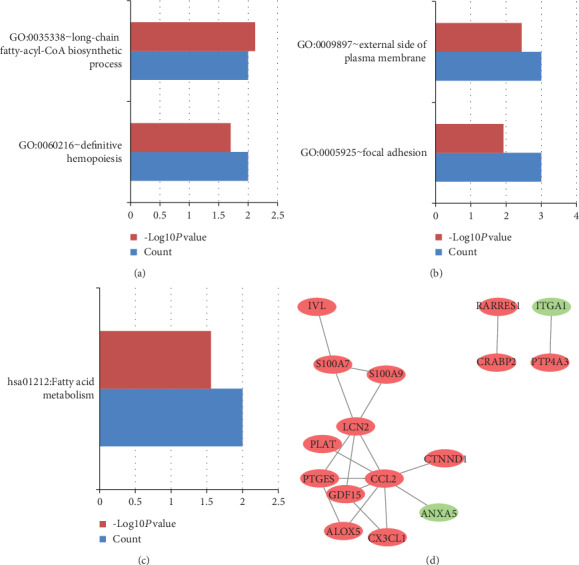
GO and KEGG pathway enrichment analyses of the downregulated DEGs, and the PPI network in GSE10432 and GSE10433. (a), (b), and (c) represent BP, CC, and KEGG, respectively. (d) is on behalf of the PPI network of GSE10432 and GSE10433; red represents the upregulated genes, and green represents the downregulated genes. Count: number of DEGs.

GSE10432 and GSE10433 represented that acne and SEB-1 sebocytes treated with isotretinoin for 1 week and 72 hours, respectively. GO and KEGG pathway enrichment analyses of the consistently upregulated or downregulated DEGs in GSE10432 and GSE10433 were clustered via DAVID. Figures 2 and 3 showed the results of GO and KEGG pathway enrichment analyses. As far as the upregulated DEGs of GSE10432 and GSE10433 were concerned, the upregulated DEGs mainly participated in cell adhesion and inflammatory response in term of BP (Figure 2(a)); located in the cytoplasm and extracellular exosome in term of CC (Figure 2(b)); involved in protein binding in term of MF (Figure 2(c)); enriched in TNF signaling pathway in term of KEGG (Figure 2(d)); all details can be seen in Table S3. In the matter of the downregulated DEGs of GSE10432 and GSE10433, the downregulated DEGs mainly involved in long-chain fatty-acyl-CoA biosynthetic process and definitive hemopoiesis in term of BP (Figure 3(a)); located in the external side of the plasma membrane and focal adhesion in term of CC (Figure 3(b)); enriched in fatty acid metabolism in term of KEGG (Figure 3(c)); all details can be seen in Table S4. The PPI network of the common DEGs in GSE10432 and GSE10433 was showed in Figure 3(d), in which the hub genes included *CCL2*, *LCN2*, *S100A7*, *PTGES*, and *GDF15* (Table 1), and *CCL2* participated in TNF signaling pathway (Table S3).

### 3.3. GO and KEGG Pathway Enrichment Analyses of the DEGs in GSE11792

**Figure 4:**
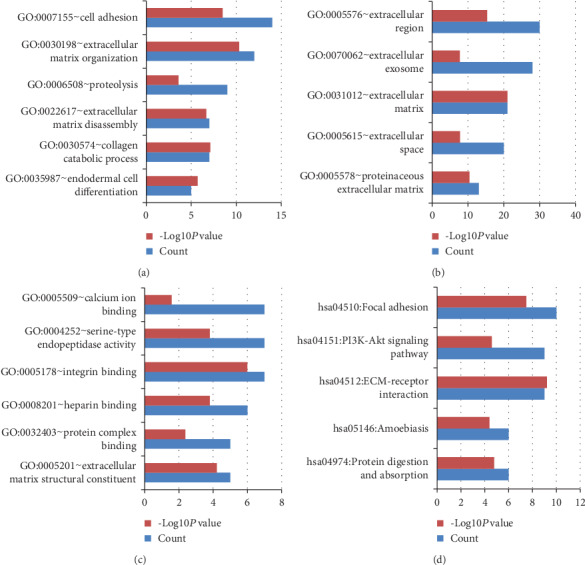
GO and KEGG pathway enrichment analyses of the upregulated DEGs in GSE11792. (a), (b), (c), and (d) represent BP, CC, MF, and KEGG, respectively. Count: number of DEGs.

**Figure 5:**
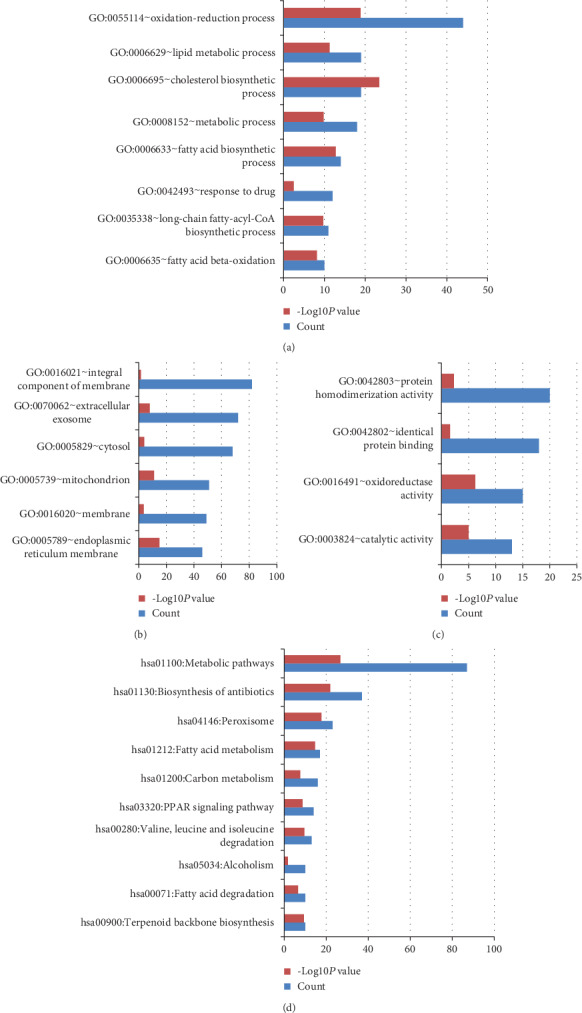
GO and KEGG pathway enrichment analyses of the downregulated DEGs in GSE11792. (a), (b), (c), and (d) represent BP, CC, MF, and KEGG, respectively. Count: number of DEGs.

GSE11792 represented that acne treated with isotretinoin for 8 weeks. GO and KEGG pathway enrichment analyses of the DEGs in GSE11792 were also clustered via DAVID. The upregulated DEGs of GSE11792 mainly involved in cell adhesion, extracellular matrix organization in term of BP (Figure 4(a)); located in the extracellular region and extracellular exosome in term of CC (Figure 4(b)); participated in integrin binding, serine-type endopeptidase activity, and calcium ion binding in term of MF (Figure 4(c)); enriched in focal adhesion, ECM-receptor interaction, and PI3K-Akt signaling pathway in term of KEGG (Figure 4(d)); all details can be seen in Table S5. The downregulated DEGs of GSE11792 mainly involved in the oxidation-reduction process, cholesterol biosynthetic process, and lipid metabolic process in term of BP (Figure 5(a)); located in integral component of the membrane and extracellular exosome in term of CC (Figure 5(b)); participated in protein homodimerization activity, identical protein binding, and catalytic activity in term of MF (Figure 5(c)); enriched in metabolic pathways, biosynthesis of antibiotics, and peroxisome in term of KEGG (Figure 5(d)); all details can be seen in Table S6.

### 3.4. PPI Network of the DEGs of GSE11792 and Identification of Hub Genes

**Figure 6:**
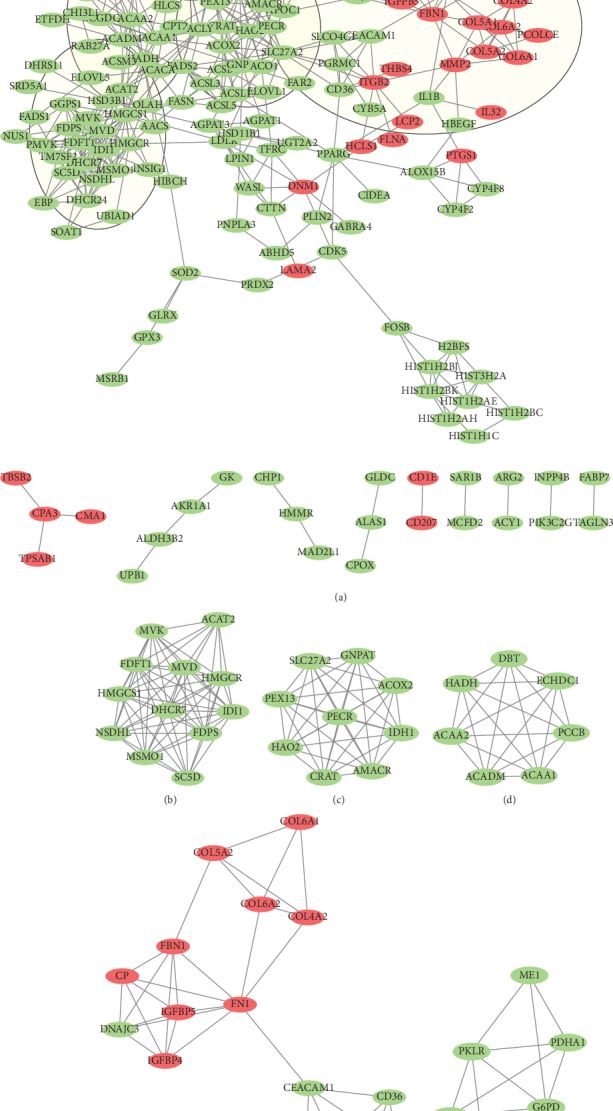
The PPI network of DEGs and module analysis in GSE11792. (a) The PPI network of GSE11792 contains 303 nodes and 478 edges; five parts of the PPI network encompassed by five circles represent five modules filtered by app MCODE in Cytoscape. (b–f) represent modules 1, 2, 3, 4, and 7, respectively, which were extracted from the PPI network. Red represents the upregulated genes, and green represents the downregulated genes.

The PPI network of the DEGs of GSE11792 was generated via STRING and Cytoscape. There were 303 nodes, including 34 upregulated and 269 downregulated DEGs, and 478 edges in the PPI network (Figure 6(a)). The term “Degree” denotes the number of interactions between two genes and was used to screen the hub genes with a threshold of Degree ≥ 10. A total of 30 hub genes were identified (Table 2), and the top 10 hub genes included *HMGCS1*, *HMGCR*, *SLC27A2*, *FDFT1*, *MVD*, *IDI1*, *IDH1*, *FDPS*, *ACAT2*, and *ACAA1*, in which all of them belonged to the downregulated DEGs of GSE11792.

### 3.5. Module Analysis of the PPI Network of GSE11792

Five modules were created from the PPI Network of GSE11792 with a cut-off point of MCODE score ≥ 4 and nodes ≥ 6, including module 1 (Score = 11.273, nodes = 12) (Figure 6(b)), module 2 (Score = 9, nodes = 9) (Figure 6(c)), module 3 (Score = 7, nodes = 7) (Figure 6(d)), module 4 (Score = 5, nodes = 15) (Figure 6(e)), and module 7 (Score = 4.8, nodes = 6) (Figure 6(f)). Most of the hub genes with Degree ≥ 10 belonged to the downregulated DEGs of GSE11792 and mainly enriched in modules 1, 2, and 3, which were mainly discussed for their KEGG pathway involved. As shown in Table 3, module 1 mainly participated in metabolic pathways and biosynthesis of antibiotics. Module 2 was involved in peroxisome and primary bile acid biosynthesis. Module 3 was mainly enriched in valine, leucine and isoleucine degradation, biosynthesis of antibiotics, metabolic pathways, and fatty acid degradation.

## 4. Discussion

There are mainly four key factors involved in the pathogenesis of acne, including follicular colonization by Propionibacterium acnes (*P. acnes*), increased sebum production, infundibular hyperkeratinization of the pilosebaceous unit, and inflammation [10]. Isotretinoin is the most potent treatment for moderate-severe acne, which targets these four mechanisms of acne pathogenesis [11]. Because of the adverse effects, especially teratogenicity of isotretinoin, multiple studies have been conducted to explore the therapeutical mechanism of isotretinoin and thus develop retinoid-like alternatives without teratogenicity in their side effects' profile. Dermcidin is an antimicrobial peptide and manifests as anti-inflammatory properties in acne vulgaris; isotretinoin can ameliorate acne vulgaris via enhancing Dermcidin level [12]. Isotretinoin increases transcription factor p53 expression, which induces expression of sestrin 1, sestrin 2, FoxO1, FoxO3, p21, and TRAIL, and suppresses levels of androgen receptor and IGF-1 receptor [13]. Studies of Nelson et al. performed microarray analyses of skin biopsies from acne patients or SEB-1 sebocytes treated with isotretinoin, explored the biological effect of lipocalin 2, which is the most highly upregulated gene [8]; however, there were other significantly upregulated or downregulated genes which might also have key functions in the acne treatment of isotretinoin. Nelson et al. also conducted GO and pathway annotations of GSE10433 and GSE11792, respectively [9], without GSE10432; also PPI networks of GSE10432, GSE10433, and GSE11792 were not constructed.

Using integrated bioinformatics analyses, there were 27 upregulated and 9 downregulated common DEGs observed in datasets of GSE10432 and GSE10433. 62 upregulated and 249 downregulated genes were found in GSE11792. The hub genes of the PPI network of the common DEGs in GSE10432 and GSE10433 included *CCL2*, *LCN2*, *S100A7*, *PTGES*, and *GDF15*; in which all of them were upregulated and *CCL2* mainly participated in TNF signaling pathway. The hub genes of PPI network of DEGs in GSE11792 included *HMGCS1*, *HMGCR*, *SLC27A2*, *FDFT1*, *MVD*, *IDI1*, *IDH1*, *FDPS*, *ACAT2*, and *ACAA1*; in which all of them were downregulated. There were mainly three modules in the PPI network of GSE11792. Module 1 mainly participated in metabolic pathways. Module 2 was mainly involved in peroxisome. Module 3 was mainly enriched in metabolic pathways and fatty acid degradation. These three modules support the effects of isotretinoin on sebum decreased production. There were only 2 DEGs including *ACSBG1* and *BCAT2*, which are downregulated among GSE10432, GSE10433, and GSE11792.

As mentioned before, the hub genes of the PPI network of the common DEGs in GSE10432 (SEB-1 sebocytes incubated with isotretinoin for 72 hours) and GSE10433 (acne patients treated with isotretinoin for 1 week) were upregulated. *CCL2*, namely, C-C Motif Chemokine Ligand 2, is a chemotactic factor which attracts monocytes and basophils but not for neutrophils or eosinophils and involves in immunoregulatory and inflammatory processes. A study of Schuster et al. showed that S1pr4 activation increases CCL2 production which promotes macrophage infiltration in a murine psoriasis model [14]. TNF signaling pathway involves in a wide range of biological processes including cell survival, apoptosis, immunity, and inflammation. A previous study showed that the aryl hydrocarbon receptor promotes secretion of inflammatory factors TNF-*α* and IL-8 in human SZ95 sebocytes, which indicates a possible mechanism in TLR2-mediated acne [15]. Compared to normal skin, the expression of TNF in acne lesions is increased; TNF also induces the formation of lipid droplets in sebocytes [16, 17]. The overexpression of *CCL2* originated from activated TNF signaling pathway and *S100A7* with chemotactic activity probably associates with some patients' side effects of “acne-flare” in early stage of isotretinoin treatment [9]. Suppression of the TNF signaling pathway and *S100A7* might be an alternative option for inhibiting “acne-flare” of acne patients while using isotretinoin. Previous studies have showed the proapoptotic effect of isotretinoin on sebocytes via mediating associated gene expression. Isotretinoin increases the levels of nuclear FoxO1 and FoxO3 proteins and participates in isotretinoin-induced proapoptotic signaling in sebocytes [18]. TRAIL is increased and involved in the apoptosis of human sebaceous gland cells mediated by isotretinoin [19]. Upregulated *LCN* expression promotes sebaceous gland cell apoptosis induced by isotretinoin [8]. In the present study, *PTGES* is a glutathione-dependent prostaglandin E synthase. Interleukin 1 beta or TP53 mediate *PTGES* expression, which probably participates in TP53 induced apoptosis. The study of Seo et al. showed that the expression of *PTGES* associates with the prognosis of colorectal cancer [20]. *GDF15* belongs to TGF-beta superfamily of proteins. A previous study showed that *GDF15* induces cytotoxicity and apoptosis in A549 cells [21]. Our study showed that the proapoptosis effect of isotretinoin on sebocytes might depend on mediating *PTGES* and *GDF15* expression except for *LCN2*.

As has been noted, the hub genes of the PPI network of DEGs in GSE11792 were downregulated, including *HMGCS1*, *HMGCR*, *FDFT1*, *MVD*, *IDI1*, and *FDPS*. All of these genes serve as critical enzymes in squalene synthesis in the biosynthetic pathway of cholesterol. Downregulated expressions of these genes were in line with the known effect of isotretinoin on decreasing sebum production. In addition, *HMGCS1* and *HMGCR* have been reported to promote cell proliferation and induce apoptosis. *HMGCS1* is upregulated and promotes cell proliferation in colon cancer tissues [22]. Overexpression of *HMGCR* is observed in human lung adenocarcinoma, and knockdown of *HMGCR* suppresses growth and promotes apoptosis of malignant cells [23]. Study of Ashida et al. showed that the knockdown of endogenous *HMGCS1* or *HMGCR* in prostate cancer (PC) cells significantly decreases PC cell viability [24]. *FDFT1* is a membrane-associated enzyme with the ability of stimulating cell growth which associates with disease development and progression in some tumors [25]. This study demonstrated that isotretinoin might also induce apoptosis of sebocytes via mediating *HMGCS1*, *HMGCR*, and *FDFT1* expression besides *LCN2*.

There were only two genes in common among datasets of GSE10432, GSE10433, and GSE11792, indicating that the effects of isotretinoin on gene expression are time-dependent in most cases. These two genes were *ACSBG1* and *BCAT2* which were downregulated. *ACSBG1* involves in S1P metabolism, which functions as a bioactive lipid molecule [26]. *BCAT2* plays an important role in the production of the branched-chain amino acids; the study showed that the depletion of *BCAT2* enzyme impairs myoblast survival, indicating a suppressing apoptosis property of *BCAT2* [27]. Isotretinoin induced downregulated expression of *ACSBG1* and *BCAT2* might be related to decreased sebum production and apoptosis of sebocytes, respectively.

In conclusion, this study exhibits that the upregulated expression of *LCN2*, *PTGES*, and *GDF15* might mediate sebocytes apoptosis and thus decreased sebum production; while *CCL2* originated from activated TNF signaling pathway and *S100A7* could be related with “acne-flare” using isotretinoin for 1 week. Downregulated genes, including *HMGCS1*, *HMGCR*, *FDFT1*, *MVD*, *IDI1*, and *FDPS* may be associated with decreased sebum synthesis; *HMGCS1*, *HMGCR*, and *FDFT1* also probably associated with apoptosis of sebocytes treated with isotretinoin for 8 weeks. *ACSBG1* and *BCAT2* work in both 1 week and 8 weeks and could associate with decreased sebum synthesis and apoptosis of sebocytes, respectively. These results indicate potential therapeutic mechanisms in the treatment of acne using isotretinoin; provide a possible target for avoiding side effect of “acne-flare”. Microarray data from acne treated with isotretinoin for 20 weeks will be helpful to investigate the mechanism of long-term remission of acne induced by isotretinoin.

## 5. Conclusions

Based on microarray datasets GSE10432, GSE10433, and GSE11792, upregulated expression of *LCN2*, *PTGES*, and *GDF15* might mediate sebocytes apoptosis; *CCL2* and *S100A7* could be related with “acne-flare” using isotretinoin for 1 week. Downregulated genes, including *HMGCS1*, *HMGCR*, *FDFT1*, *MVD*, *IDI1*, and *FDPS* may be associated with decreased sebum synthesis; *HMGCS1*, *HMGCR*, and *FDFT1* also probably associate with the apoptosis of sebocytes treated with isotretinoin for 8 weeks. *ACSBG1* and *BCAT2* work in both 1 week and 8 weeks and could associate with decreased sebum synthesis and apoptosis of sebocytes, respectively.

## Figures and Tables

**Table 1:** The hub genes in the PPI network of GSE10432 and GSE10433.

ID	Degree	ID	Degree	ID	Degree	ID	Degree
*CCL2*	8	*GDF15*	3	*PTP4A3*	1	*CRABP2*	1
*LCN2*	5	*CX3CL1*	2	*ITGA1*	1	*RARRES1*	1
*S100A7*	3	*ALOX5*	2	*IVL*	1	*PLAT*	1
*PTGES*	3	*S100A9*	2	*ANXA5*	1	*CTNND1*	1

Note: Degree: the number of interactions between two genes in the PPI network. Abbreviations: PPI: protein–protein interaction.

**Table 2:** The hub genes in the PPI network of GSE11792.

ID	Degree	ID	Degree	ID	Degree	ID	Degree
*HMGCS1*	21	*ACAT2*	15	*MSMO1*	13	*TM7SF2*	10
*HMGCR*	18	*ACAA1*	15	*GNPAT*	13	*PC*	10
*SLC27A2*	17	*FN1*	14	*ACOX2*	13	*G6PD*	10
*FDFT1*	17	*DHCR7*	14	*ACLY*	13	*FBN1*	10
*MVD*	15	*SC5D*	13	*ACAA2*	12	*DHCR24*	10
*IDI1*	15	*PEX13*	13	*HADH*	11	*CRAT*	10
*IDH1*	15	*NSDHL*	13	*DBT*	11		
*FDPS*	15	*MVK*	13	*ACADM*	11		

Note: Degree: the number of interactions between two genes in the PPI network. Abbreviations: PPI: protein–protein interaction.

**Table 3:** KEGG pathway enrichment analysis of genes of modules 1, 2, and 3 in the PPI network of GSE11792.

Term	Description	Count	*P* value	Genes
Module 1				
hsa01100	Metabolic pathways	12	5.21*E*-09	*SC5D*, *MSMO1*, *MVD*, *HMGCR*, *DHCR7*, *FDPS*, *HMGCS1*, *MVK*, *ACAT2*, *IDI1*, *NSDHL*, *FDFT1*
hsa01130	Biosynthesis of antibiotics	11	6.70*E*-15	*SC5D*, *MSMO1*, *MVD*, *HMGCR*, *FDPS*, *HMGCS1*, *MVK*, *ACAT2*, *IDI1*, *NSDHL*, *FDFT1*
hsa00900	Terpenoid backbone biosynthesis	7	2.32*E*-13	*MVD*, *HMGCR*, *FDPS*, *HMGCS1*, *MVK*, *ACAT2*, *IDI1*
hsa00100	Steroid biosynthesis	5	1.69*E*-08	*SC5D*, *MSMO1*, *DHCR7*, *NSDHL*, *FDFT1*
hsa00072	Synthesis and degradation of ketone bodies	2	0.015886	*HMGCS1*, *ACAT2*
hsa00650	Butanoate metabolism	2	0.042368	*HMGCS1*, *ACAT2*
Module 2				
hsa04146	Peroxisome	9	3.18*E*-16	*ACOX2*, *PECR*, *AMACR*, *HAO2*, *GNPAT*, *IDH1*, *PEX13*, *CRAT*, *SLC27A2*
hsa00120	Primary bile acid biosynthesis	2	0.01961	*ACOX2*, *AMACR*
Module 3				
hsa00280	Valine, leucine and isoleucine degradation	6	7.14*E*-11	*ACAA2*, *DBT*, *ACADM*, *HADH*, *PCCB*, *ACAA1*
hsa01130	Biosynthesis of antibiotics	6	1.55*E*-07	*ACAA2*, *DBT*, *ACADM*, *HADH*, *PCCB*, *ACAA1*
hsa01100	Metabolic pathways	6	8.88*E*-04	*ACAA2*, *DBT*, *ACADM*, *HADH*, *PCCB*, *ACAA1*
hsa00071	Fatty acid degradation	4	4.18*E*-06	*ACAA2*, *ACADM*, *HADH*, *ACAA1*
hsa01212	Fatty acid metabolism	4	6.29*E*-06	*ACAA2*, *ACADM*, *HADH*, *ACAA1*
hsa00640	Propanoate metabolism	3	2.37E-04	*ACADM*, *ECHDC1*, *PCCB*
hsa00062	Fatty acid elongation	2	0.021616	*ACAA2*, *HADH*

Notes: Count: the number of DEGs. Abbreviations: DEGs: differentially expressed genes; KEGG: Kyoto Encyclopedia of Genes and Genomes; PPI: protein–protein interaction.

## Data Availability

The data used to support the findings of this study are included within the article and the supplementary information files.

## Supplementary Materials

Supplementary Materials . Table S1: 36 DEGs were extracted from 2 microarray data GSE10432 and GSE10433, including 27 upregulated genes and 9 downregulated genes. Table S2: 62 upregulated genes and 249 downregulated genes observed in GSE11792. Table S3: GO and KEGG pathway enrichment analyses for the common upregulated DEGs of GSE10432 and GSE10433. Table S4: GO and KEGG pathway enrichment analyses for the common downregulated DEGs of GSE10432 and GSE10433. Table S5: GO and KEGG pathway enrichment analyses for the upregulated DEGs of GSE11792. Table S6: GO and KEGG pathway enrichment analyses for the downregulated DEGs of GSE11792.

## References

[1] Tan J. K. L., Bhate K. (2015). A global perspective on the epidemiology of acne. *The British Journal of Dermatology*.

[2] Barnes L. E., Levender M. M., Fleischer A. B., Feldman S. R. (2012). Quality of life measures for acne patients. *Dermatologic Clinics*.

[3] Zouboulis C. C., Dessinioti C., Tsatsou F., Gollnick H. P. M. (2017). Anti-acne drugs in phase 1 and 2 clinical trials. *Expert Opinion on Investigational Drugs*.

[4] Zaenglein A. L., Pathy A. L., Schlosser B. J. (2016). Guidelines of care for the management of acne vulgaris. *Journal of the American Academy of Dermatology*.

[5] Nast A., Dreno B., Bettoli V. (2012). European evidence-based (S3) guidelines for the treatment of acne. *Journal of the European Academy of Dermatology and Venereology*.

[6] Pona A., la Garza J. A. C.-d., Haidari W., Cline A., Feldman S. R., Taylor S. L. (2019). Abnormal liver function tests in acne patients receiving isotretinoin. *Journal of Dermatological Treatment*.

[7] Vallerand I. A., Lewinson R. T., Farris M. S. (2018). Efficacy and adverse events of oral isotretinoin for acne: a systematic review. *British Journal of Dermatology*.

[8] Nelson A. M., Zhao W., Gilliland K. L., Zaenglein A. L., Liu W., Thiboutot D. M. (2008). Neutrophil gelatinase-associated lipocalin mediates 13-cis retinoic acid-induced apoptosis of human sebaceous gland cells. *The Journal of Clinical Investigation*.

[9] Nelson A. M., Zhao W., Gilliland K. L., Zaenglein A. L., Liu W., Thiboutot D. M. (2009). Isotretinoin temporally regulates distinct sets of genes in patient skin. *The Journal of Investigative Dermatology*.

[10] Chen B., Zheng Y., Liang Y. (2019). Analysis of potential genes and pathways involved in the pathogenesis of acne by Bioinformatics. *BioMed Research International*.

[11] Tuchayi S. M., Makrantonaki E., Ganceviciene R., Dessinioti C., Feldman S. R., Zouboulis C. C. (2015). Acne vulgaris. *Nature Reviews. Disease Primers*.

[12] Alatas E. T., Kara Polat A., Kalayci M., Dogan G., Akin Belli A. (2019). Plasma dermcidin levels in acne patients, and the effect of isotretinoin treatment on dermcidin levels. *Dermatologic Therapy*.

[13] Melnik B. C. (2017). p53: key conductor of all anti-acne therapies. *Journal of Translational Medicine*.

[14] Schuster C., Huard A., Sirait-Fischer E., Dillmann C., Brune B., Weigert A. (2020). S1PR4-dependent CCL2 production promotes macrophage recruitment in a muine psoriasis model. *European Journal of Immunology*.

[15] Hou X. X., Chen G., Hossini A. M. (2018). Aryl hydrocarbon receptor modulates the expression of TNF-*α* and IL-8 in human sebocytes via the MyD88-p65NF-*κ*B/p38MAPK signaling pathways. *Journal of Innate Immunity*.

[16] Liu J., Cao L., Feng Y., Li Y., Li T. (2017). MiR-338-3p inhibits TNF-*α*-induced lipogenesis in human sebocytes. *Biotechnology Letters*.

[17] Choi J. J., Park M. Y., Lee H. J. (2012). TNF-*α* increases lipogenesis via JNK and PI3K/Akt pathways in SZ95 human sebocytes. *Journal of Dermatological Science*.

[18] Agamia N. F., Roshdy O. H., Abdelmaksoud R. E. S. (2018). Effect of oral isotretinoin on the nucleo-cytoplasmic distribution of FoxO1 and FoxO3 proteins in sebaceous glands of patients with acne vulgaris. *Experimental Dermatology*.

[19] Nelson A. M., Cong Z., Gilliland K. L., Thiboutot D. M. (2011). TRAIL contributes to the apoptotic effect of 13-cis retinoic acid in human sebaceous gland cells. *The British Journal of Dermatology*.

[20] Seo T., Tatsuguchi A., Shinji S. (2009). Microsomal prostaglandin E synthase protein levels correlate with prognosis in colorectal cancer patients. *Virchows Archiv*.

[21] Tarfiei G. A., Shadboorestan A., Montazeri H., Rahmanian N., Tavosi G., Ghahremani M. H. (2019). GDF15 induced apoptosis and cytotoxicity in A549 cells depends on TGFBR2 expression. *Cell Biochemistry and Function*.

[22] Zhou S., Xu H., Tang Q., Xia H., Bi F. (2020). Dipyridamole enhances the cytotoxicities of trametinib against colon cancer cells through combined targeting of HMGCS1 and MEK pathway. *Molecular Cancer Therapeutics*.

[23] Zhang T., Bai R., Wang Q. (2019). Fluvastatin inhibits HMG-CoA reductase and prevents non-small cell lung carcinogenesis. *Cancer Prevention Research*.

[24] Ashida S., Kawada C., Inoue K. (2017). Stromal regulation of prostate cancer cell growth by mevalonate pathway enzymes HMGCS1 and HMGCR. *Oncology Letters*.

[25] Tuzmen S., Hostetter G., Watanabe A. (2019). Characterization of farnesyl diphosphate farnesyl transferase 1 (FDFT1) expression in cancer. *Personalized Medicine*.

[26] Ohkuni A., Ohno Y., Kihara A. (2013). Identification of acyl-CoA synthetases involved in the mammalian sphingosine 1-phosphate metabolic pathway. *Biochemical and Biophysical Research Communications*.

[27] Dhanani Z. N., Mann G., Adegoke O. A. J. (2019). Depletion of branched-chain aminotransferase 2 (BCAT2) enzyme impairs myoblast survival and myotube formation. *Physiological Reports*.

