# Supplementary material for: Putative Genes and Pathways Involved in the Acne Treatment of Isotretinoin via Microarray Data Analyses
Source: Biomed Res Int. 2020 Jun 29;2020:5842795. doi: 10.1155/2020/5842795 (PMC7341380; doi:10.1155/2020/5842795)
Supplement: Supplementary Materials — . Table S1: 36 DEGs were extracted from 2 microarray data GSE10432 and GSE10433, including 27 upregulated genes and 9 downregulated genes. Table S2: 62 upregulated genes and 249 downregulated genes observed in GSE11792. Table S3: GO and KEGG pathway enrichment analyses for the common upregulated DEGs of GSE10432 and GSE10433. Table S4: GO and KEGG pathway enrichment analyses for the common downregulated DEGs of GSE10432 and GSE10433. Table S5: GO and KEGG pathway enrichment analyses for the upregulated DEGs of GSE11792. Table S6: GO and KEGG pathway enrichment analyses for the downregulated DEGs of GSE11792. [file 5842795.f1.docx]

**Table S1**. 36 DEGs were extracted from 2 microarray data GSE10432 and GSE10433, including 27 upregulated genes and 9 downregulated genes.

| **DEGs** | **Gene symbol** |
| --- | --- |
| **Up-regulated**  **genes** | *RARRES1, LCN2, CEACAM1, GDF15, ANXA3, ELF3, TTC9, S100A7, S100A9, EPB41L1, IVL, EPHB2, BLNK, CRABP2, MAGI1, TRIM16, HK2, SKAP1, ALOX5, CTNND1, CCL2, PLAT, TNFRSF6B, PTGES, PTP4A3, BCL3, CX3CL1* |
| **Down-regulated genes** | *ACSBG1, BAG2, BCAT2, ITGA1, TGFBR3, MFAP5, HACD2, CSRP2, ANXA5* |

**Abbreviations**: DEGs, differentially expressed genes.

**Table S2**. 62 upregulated genes and 249 downregulated genes observed in GSE11792.

| **DEGs** | **Gene symbol** |
| --- | --- |
| **Up-regulated**  **genes** | *MSMB, CAPN6, COCH, LCP2, FBLN1, CD1E, SERHL2, SUSD5, CPA3, PTGS1, COL6A2, BTN3A3, TPSAB1, MFAP4, COTL1, CTSK, RHOBTB3, TPSB2, WIF1, SLIT3, CX3CR1, MFAP2, SERHL2, ITIH5, PCOLCE, TNN, DPT, MMP2, PLEC, FHL1, FLNA, ABCA8, FN1, CD207, PPP1R16B, CP, HTRA1, COL5A1, POGZ, SAMSN1, COL6A1, COL5A2, IGFBP4, SNED1, ITGBL1, HCLS1, CHL1, LAMA2, ITGB2, COL4A2, CMA1, TPSB2, FBN1, FBLN5, C1S, PTPRG, MN1, IL32, THBS4, IGFBP5, SERPING1, DNM1* |
| **Down-regulated genes** | *MCFD2, GLRX, RAB27A, HIST3H2A, DHFR, C20orf24, FAM134B, PAQR3, MAD2L1, ACSL3, AGPAT1, DNAJC3, MPC2, HADH, RIDA, GGPS1, HIBCH, HIST1H2AH, PIR, SPCS3, SLC50A1, C21orf33, GNG4, PIK3C2G, INPP4B, APOOL, SLC7A5, ELOVL1, DHCR24, PGRMC1, ZNF675, SLC16A7, CTTN, ISOC1, HBEGF, CTSV, CPOX, CDA, CLSTN3, PPIF, ULK4, C9orf16, HLCS, AKR1A1, AMACR, ZNF385D, FOSB, PMEL, PEX16, IER3, TAGLN3, RBM47, MPC1, ENDOU, IL1B, NTAN1, PC, ACACA, ANKRD12, AGR2, CDK5, WASL, LDLR, TFRC, PPARG, CD36, DCXR, SOD2, TMEM254, SNORD73A, HMMR, NUS1P3, LRRD1, ZNF165, CYP4F2, CKMT1A, PRDX2, DSC2, ACSL5, GK6P, CALML3, KRT9, DBT, TSPAN6, DOPEY2, SLC15A1, TST, ALAS1, SAR1B, LPIN1, SLC25A16, ACLY, NADK, H2BFS, SORD, ACY1, HIST1H2BK, PEX3, FKBP5, SLC31A1, PEX13, E2F8, TMEM164, ETFDH, GNPAT, SLC30A10, CHI3L2, SNX13, SYN2, ZNF43, IDH1, SLC46A3, CPT2, AACS, ECHDC1, PGD, CERS4, BEAN1, ACAA1, NOP16, PEX11A, FDFT1, ZNF254, ZNF91, OAS1, DHRS11, DIRAS3, PDHA1, GABRA4, ACO1, ELOVL4, CEACAM1, TKT, NUS1, PHLDA2, SC5D, KCNJ15, G6PD, MVK, AGPAT3, SLC25A17, CHP1, IDI1, MFAP3L, PXMP2, PNPLA3, ZNF729, HIST1H2BJ, GPX3, PXMP4, HIST1H2AE, MSRB1, DUSP4, PMVK, PCCB, ALDH3B2, DDC, ACAD8, SGK2, TM7SF2, CYP4F2, BCKDHB, SNORA29, ARG2, CTH, MOCOS, CYB5A, ACOX2, ACP6, ADGRG2, ELOVL5, PCTP, EBP, NPL, RDH11, PLIN2, BCAT2, APMAP, SAA2, NSDHL, ACSL1, ZBTB16, PON3, MECR, DHCR7, MVD, ACAA2, MC5R, GK3P, OTUB2, DNASE1L2, TMED5, ACADM, FASN, ADTRP, HIST1H2BC, ABHD5, BCAP29, MSMO1, GK, HMGCR, SLC26A3, FBP1, UPB1, HILPDA, CUX2, FDPS, ACAT2, UGT2A2, ME1, PKLR, FA2H, CYP4F8, CIDEA, PDE6A, SEC14L4, TMPRSS11E, HSD11B1, SRD5A1, SLC27A2, ACSM3, HGD, HMGCS1, PECR, APOC1, UBIAD1, CHI3L1, DHRS9, ACSBG1, ACOT1, TMEM97, CRAT, SOAT1, HIST1H1C, MUC1, FADS2, INSIG1, ALOX15B, FABP7, PLA2G7, GAL, PDZK1, SLCO4C1, FAR2, FADS1, GLDC, HAO2, OLAH, HSD3B1* |

**Abbreviations**: DEGs, differentially expressed genes.

**Table S3.** GO and KEGG pathway enrichment analyses for the common upregulated DEGs of GSE10432 and GSE10433.

| **Category** | **Term** | **Description** | **Count** | ***p*-Value** | **Genes** |
| --- | --- | --- | --- | --- | --- |
| BP | GO:0006954 | inflammatory response | 5 | 0.002577 | *TNFRSF6B, CCL2, ELF3, S100A9, BLNK* |
| BP | GO:0007155 | cell adhesion | 5 | 0.005111 | *CCL2, MAGI1, CTNND1, CX3CL1, CEACAM1* |
| BP | GO:0001525 | angiogenesis | 4 | 0.004795 | *CCL2, S100A7, CEACAM1, EPHB2* |
| BP | GO:0045893 | positive regulation of transcription, DNA-templated | 4 | 0.044214 | *ELF3, BCL3, TRIM16, SKAP1* |
| BP | GO:0002523 | leukocyte migration involved in inflammatory response | 3 | 1.26E-04 | *CCL2, S100A9, CX3CL1* |
| BP | GO:0030593 | neutrophil chemotaxis | 3 | 0.004653 | *CCL2, S100A9, CX3CL1* |
| BP | GO:0071347 | cellular response to interleukin-1 | 3 | 0.005365 | *LCN2, CCL2, CX3CL1* |
| BP | GO:0050729 | positive regulation of inflammatory response | 3 | 0.005663 | *CCL2, S100A9, CX3CL1* |
| BP | GO:0008544 | epidermis development | 3 | 0.007606 | *ELF3, S100A7, CRABP2* |
| BP | GO:0071356 | cellular response to tumor necrosis factor | 3 | 0.012473 | *LCN2, CCL2, CX3CL1* |
| BP | GO:0042742 | defense response to bacterium | 3 | 0.021016 | *S100A9, BCL3, ANXA3* |
| BP | GO:0032496 | response to lipopolysaccharide | 3 | 0.026429 | *TNFRSF6B, S100A7, PTGES* |
| BP | GO:0070374 | positive regulation of ERK1 and ERK2 cascade | 3 | 0.029794 | *CCL2, S100A7, CX3CL1* |
| BP | GO:0048246 | macrophage chemotaxis | 2 | 0.01995 | *CCL2, CX3CL1* |
| BP | GO:0090026 | positive regulation of monocyte chemotaxis | 2 | 0.024499 | *CCL2, S100A7* |
| BP | GO:0048247 | lymphocyte chemotaxis | 2 | 0.042493 | *CCL2, CX3CL1* |
| CC | GO:0005737 | cytoplasm | 15 | 0.006036 | *PLAT, ELF3, MAGI1, S100A7, CRABP2, CTNND1, TRIM16, SKAP1, ANXA3, EPB41L1, PTP4A3, BCL3, GDF15, IVL, BLNK* |
| CC | GO:0070062 | extracellular exosome | 11 | 0.003693 | *PLAT, LCN2, RARRES1, S100A7, CRABP2, S100A9, CTNND1, GDF15, IVL, CEACAM1, ANXA3* |
| CC | GO:0005829 | cytosol | 11 | 0.012159 | *LCN2, EPB41L1, S100A7, CRABP2, S100A9, HK2, CTNND1, ALOX5, SKAP1, BLNK, EPHB2* |
| CC | GO:0005576 | extracellular region | 9 | 0.001337 | *PLAT, LCN2, TNFRSF6B, CCL2, S100A7, S100A9, CX3CL1, GDF15, EPHB2* |
| CC | GO:0005615 | extracellular space | 8 | 0.002237 | *PLAT, LCN2, TNFRSF6B, CCL2, S100A9, ALOX5, CX3CL1, GDF15* |
| CC | GO:0005911 | cell-cell junction | 5 | 9.74E-05 | *EPB41L1, MAGI1, CTNND1, SKAP1, CEACAM1* |
| CC | GO:0005641 | nuclear envelope lumen | 2 | 0.011359 | *PTGES, ALOX5* |
| CC | GO:0042101 | T cell receptor complex | 2 | 0.025383 | *SKAP1, CEACAM1* |
| MF | GO:0005515 | protein binding | 20 | 0.012507 | *TNFRSF6B, PLAT, ELF3, MAGI1, S100A7, CRABP2, S100A9, HK2, CTNND1, TRIM16, CX3CL1, SKAP1, EPHB2, EPB41L1, BCL3, ALOX5, GDF15, IVL, CEACAM1, BLNK* |
| MF | GO:0005102 | receptor binding | 5 | 0.00168 | *PLAT, CCL2, CTNND1, CX3CL1, EPHB2* |
| MF | GO:0050786 | RAGE receptor binding | 2 | 0.016175 | *S100A7, S100A9* |
| **KEGG** | **hsa04668** | **TNF signaling pathway** | **3** | **0.014291** | ***CCL2, BCL3, CX3CL1*** |

**Notes**: Count, the number of DEGs.

**Abbreviations**: GO, Gene Ontology; KEGG, Kyoto Encyclopedia of Genes and Genomes; DEGs, differentially expressed genes; MF, molecular function; CC, cellular component; BP, biological process.

**Table S4.** GO and KEGG pathway enrichment analyses for the common downregulated DEGs of GSE10432 and GSE10433.

| **Category** | **Term** | **Description** | **Count** | ***p*-Value** | **Genes** |
| --- | --- | --- | --- | --- | --- |
| BP | GO:0060216 | definitive hemopoiesis | 2 | 0.007599 | *TGFBR3, MFAP5* |
| BP | GO:0035338 | long-chain fatty-acyl-CoA biosynthetic process | 2 | 0.019839 | *HACD2, ACSBG1* |
| CC | GO:0009897 | external side of plasma membrane | 3 | 0.003635 | *ITGA1, TGFBR3, ANXA5* |
| CC | GO:0005925 | focal adhesion | 3 | 0.011802 | *ITGA1, CSRP2, ANXA5* |
| MF | GO:0003824 | catalytic activity | 2 | 0.085715 | *BCAT2, ACSBG1* |
| **KEGG** | **hsa01212** | **Fatty acid metabolism** | **2** | **0.027626** | ***HACD2, ACSBG1*** |

**Notes**: Count, the number of DEGs.

**Abbreviations**: GO, Gene Ontology; KEGG, Kyoto Encyclopedia of Genes and Genomes; DEGs, differentially expressed genes; MF, molecular function; CC, cellular component; BP, biological process.

**Table S5.** GO and KEGG pathway enrichment analyses for the common upregulated DEGs of GSE11792.

| **Category** | **Term** | **Description** | **Count** | ***p*-Value** | **Genes** |
| --- | --- | --- | --- | --- | --- |
| BP | GO:0007155 | cell adhesion | 14 | 3.29E-09 | *ITGB2, IL32, COL5A1, ITGBL1, LAMA2, CX3CR1, COL6A2, SUSD5, COL6A1, MFAP4, CHL1, FN1, THBS4, DPT* |
| BP | GO:0030198 | extracellular matrix organization | 12 | 4.78E-11 | *LAMA2, COL4A2, FBLN1, FBLN5, FBN1, COL6A2, COL6A1, MFAP2, ITGB2, COL5A2, COL5A1, FN1* |
| BP | GO:0006508 | proteolysis | 9 | 2.68E-04 | *CAPN6, CTSK, HTRA1, CPA3, C1S, TPSB2, TPSAB1, MMP2, PCOLCE* |
| BP | GO:0030574 | collagen catabolic process | 7 | 7.52E-08 | *COL4A2, CTSK, COL6A2, COL6A1, MMP2, COL5A2, COL5A1* |
| BP | GO:0022617 | extracellular matrix disassembly | 7 | 2.12E-07 | *CTSK, HTRA1, FBN1, CMA1, TPSAB1, MMP2, FN1* |
| BP | GO:0035987 | endodermal cell differentiation | 5 | 1.98E-06 | *COL4A2, COL6A1, ITGB2, MMP2, FN1* |
| BP | GO:0001558 | regulation of cell growth | 4 | 0.002532 | *HTRA1, FBLN5, IGFBP4, IGFBP5* |
| BP | GO:0007160 | cell-matrix adhesion | 4 | 0.003535 | *SNED1, FBLN5, TNN, ITGB2* |
| BP | GO:0001957 | intramembranous ossification | 3 | 1.68E-04 | *CTSK, MN1, MMP2* |
| BP | GO:0010952 | positive regulation of peptidase activity | 3 | 8.62E-04 | *FBLN1, PCOLCE, FN1* |
| BP | GO:0030199 | collagen fibril organization | 3 | 0.00774 | *COL5A2, COL5A1, DPT* |
| BP | GO:0071230 | cellular response to amino acid stimulus | 3 | 0.011097 | *COL6A1, MMP2, COL5A2* |
| BP | GO:0071560 | cellular response to transforming growth factor beta stimulus | 3 | 0.01202 | *COL4A2, FBN1, CX3CR1* |
| BP | GO:0016525 | negative regulation of angiogenesis | 3 | 0.018793 | *COL4A2, CX3CR1, THBS4* |
| BP | GO:0007229 | integrin-mediated signaling pathway | 3 | 0.04453 | *FBLN1, ITGB2, ITGBL1* |
| BP | GO:0002576 | platelet degranulation | 3 | 0.04781 | *SERPING1, FLNA, FN1* |
| BP | GO:1903225 | negative regulation of endodermal cell differentiation | 2 | 0.006778 | *COL5A2, COL5A1* |
| BP | GO:1904237 | positive regulation of substrate-dependent cell migration, cell attachment to substrate | 2 | 0.006778 | *FBLN1, FN1* |
| BP | GO:2001202 | negative regulation of transforming growth factor-beta secretion | 2 | 0.010149 | *FBLN1, FN1* |
| BP | GO:0048592 | eye morphogenesis | 2 | 0.010149 | *COL5A2, COL5A1* |
| BP | GO:0048050 | post-embryonic eye morphogenesis | 2 | 0.01351 | *FBN1, MFAP2* |
| BP | GO:0048251 | elastic fiber assembly | 2 | 0.023525 | *FBLN5, MFAP4* |
| BP | GO:0044342 | type B pancreatic cell proliferation | 2 | 0.030146 | *IGFBP4, IGFBP5* |
| BP | GO:0048048 | embryonic eye morphogenesis | 2 | 0.03344 | *FBN1, MFAP2* |
| BP | GO:0002003 | angiotensin maturation | 2 | 0.036723 | *CPA3, CMA1* |
| BP | GO:0043206 | extracellular fibril organization | 2 | 0.039995 | *MFAP4, COL5A1* |
| BP | GO:0043568 | positive regulation of insulin-like growth factor receptor signaling pathway | 2 | 0.043256 | *IGFBP4, IGFBP5* |
| BP | GO:0070208 | protein heterotrimerization | 2 | 0.046506 | *COL6A2, COL6A1* |
| BP | GO:0042993 | positive regulation of transcription factor import into nucleus | 2 | 0.049745 | *HCLS1, FLNA* |
| CC | GO:0005576 | extracellular region | 30 | 4.69E-16 | *C1S, MMP2, ITGBL1, HTRA1, COL6A2, COL6A1, CPA3, ITIH5, TPSB2, THBS4, FN1, COL4A2, FBN1, SERPING1, COL5A2, COL5A1, FLNA, SLIT3, LAMA2, CTSK, FBLN1, FBLN5, CMA1, WIF1, MFAP2, CP, TPSAB1, MFAP4, IGFBP4, IGFBP5* |
| CC | GO:0070062 | extracellular exosome | 28 | 2.06E-08 | *PTGS1, ITGB2, C1S, PCOLCE, HTRA1, COL6A2, COL6A1, PLEC, THBS4, RHOBTB3, FN1, DPT, COCH, COL4A2, PTPRG, FBN1, SERPING1, COTL1, COL5A1, FLNA, LAMA2, FBLN1, SNED1, FBLN5, CP, MFAP4, DNM1, CHL1* |
| CC | GO:0031012 | extracellular matrix | 21 | 8.84E-22 | *COCH, COL4A2, FBN1, COL5A2, MMP2, PCOLCE, FLNA, COL5A1, LAMA2, FBLN1, HTRA1, FBLN5, COL6A2, COL6A1, CMA1, MFAP4, TPSAB1, PLEC, FN1, THBS4, DPT* |
| CC | GO:0005615 | extracellular space | 20 | 1.65E-08 | *PTPRG, MSMB, FBN1, IL32, SERPING1, MMP2, PCOLCE, SLIT3, CTSK, FBLN1, HTRA1, FBLN5, COL6A2, CPA3, CP, TPSAB1, IGFBP4, FN1, THBS4, DPT* |
| CC | GO:0005578 | proteinaceous extracellular matrix | 13 | 3.91E-11 | *COCH, FBLN1, FBLN5, FBN1, COL6A2, TNN, MMP2, COL5A2, CHL1, COL5A1, FN1, SLIT3, DPT* |
| CC | GO:0005604 | basement membrane | 5 | 1.16E-04 | *LAMA2, FBLN1, FBN1, COL5A1, THBS4* |
| CC | GO:0005581 | collagen trimer | 5 | 2.09E-04 | *COL6A2, COL6A1, COL5A2, PCOLCE, COL5A1* |
| CC | GO:0005788 | endoplasmic reticulum lumen | 5 | 0.003251 | *COL4A2, COL6A2, COL6A1, COL5A2, COL5A1* |
| CC | GO:0042383 | sarcolemma | 4 | 0.002511 | *LAMA2, COL6A2, COL6A1, PLEC* |
| CC | GO:0072562 | blood microparticle | 4 | 0.012559 | *SERPING1, C1S, CP, FN1* |
| CC | GO:0071953 | elastic fiber | 3 | 5.95E-05 | *FBLN1, FBLN5, MFAP4* |
| CC | GO:0001527 | microfibril | 3 | 4.41E-04 | *FBN1, MFAP2, MFAP4* |
| CC | GO:0005588 | collagen type V trimer | 2 | 0.009518 | *COL5A2, COL5A1* |
| CC | GO:0005577 | fibrinogen complex | 2 | 0.028288 | *FBLN1, FN1* |
| CC | GO:0005605 | basal lamina | 2 | 0.046708 | *LAMA2, FN1* |
| MF | GO:0005178 | integrin binding | 7 | 1.02E-06 | *FBLN1, FBLN5, FBN1, TNN, COL5A1, FN1, THBS4* |
| MF | GO:0004252 | serine-type endopeptidase activity | 7 | 1.58E-04 | *CTSK, HTRA1, CMA1, C1S, TPSB2, TPSAB1, MMP2* |
| MF | GO:0005509 | calcium ion binding | 7 | 0.026325 | *FBLN1, SNED1, FBLN5, FBN1, C1S, THBS4, SLIT3* |
| MF | GO:0008201 | heparin binding | 6 | 1.56E-04 | *FBN1, PCOLCE, COL5A1, FN1, THBS4, SLIT3* |
| MF | GO:0005201 | extracellular matrix structural constituent | 5 | 6.17E-05 | *COL4A2, FBLN1, FBN1, COL5A2, COL5A1* |
| MF | GO:0032403 | protein complex binding | 5 | 0.00423 | *FBLN1, HCLS1, FBN1, ITGB2, DNM1* |
| MF | GO:0005518 | collagen binding | 4 | 9.31E-04 | *COCH, CTSK, PCOLCE, FN1* |
| MF | GO:0008236 | serine-type peptidase activity | 4 | 0.001073 | *HTRA1, CMA1, TPSB2, TPSAB1* |
| MF | GO:0016504 | peptidase activator activity | 3 | 4.45E-04 | *FBLN1, PCOLCE, FN1* |
| MF | GO:0001968 | fibronectin binding | 3 | 0.003107 | *CTSK, FBLN1, IGFBP5* |
| MF | GO:0031995 | insulin-like growth factor II binding | 2 | 0.025311 | *IGFBP4, IGFBP5* |
| MF | GO:0048407 | platelet-derived growth factor binding | 2 | 0.03464 | *COL6A1, COL5A1* |
| MF | GO:0043394 | proteoglycan binding | 2 | 0.03464 | *CTSK, COL5A1* |
| MF | GO:0031994 | insulin-like growth factor I binding | 2 | 0.03773 | *IGFBP4, IGFBP5* |
| KEGG | hsa04510 | Focal adhesion | 10 | 3.27E-08 | *LAMA2, COL4A2, COL6A2, COL6A1, TNN, COL5A2, FLNA, COL5A1, FN1, THBS4* |
| KEGG | hsa04512 | ECM-receptor interaction | 9 | 6.13E-10 | *LAMA2, COL4A2, COL6A2, COL6A1, TNN, COL5A2, COL5A1, FN1, THBS4* |
| KEGG | hsa04151 | PI3K-Akt signaling pathway | 9 | 2.60E-05 | *LAMA2, COL4A2, COL6A2, COL6A1, TNN, COL5A2, COL5A1, FN1, THBS4* |
| KEGG | hsa04974 | Protein digestion and absorption | 6 | 1.63E-05 | *COL4A2, COL6A2, CPA3, COL6A1, COL5A2, COL5A1* |
| KEGG | hsa05146 | Amoebiasis | 6 | 4.02E-05 | *LAMA2, COL4A2, ITGB2, COL5A2, COL5A1, FN1* |
| KEGG | hsa04611 | Platelet activation | 4 | 0.012482 | *PTGS1, COL5A2, COL5A1, LCP2* |
| KEGG | hsa05205 | Proteoglycans in cancer | 4 | 0.038508 | *HCLS1, MMP2, FLNA, FN1* |
| KEGG | hsa05133 | Pertussis | 3 | 0.032199 | *SERPING1, ITGB2, C1S* |
| KEGG | hsa05100 | Bacterial invasion of epithelial cells | 3 | 0.034605 | *HCLS1, DNM1, FN1* |
| KEGG | hsa05222 | Small cell lung cancer | 3 | 0.040484 | *LAMA2, COL4A2, FN1* |

**Notes**: Count, the number of DEGs.

**Abbreviations**: GO, Gene Ontology; KEGG, Kyoto Encyclopedia of Genes and Genomes; DEGs, differentially expressed genes; MF, molecular function; CC, cellular component; BP, biological process.

**Table S6.** GO and KEGG pathway enrichment analyses for the common downregulated DEGs of GSE11792.

| **Category** | **Term** | **Description** | **Count** | ***p*-Value** | **Genes** |
| --- | --- | --- | --- | --- | --- |
| BP | GO:0055114 | oxidation-reduction process | 44 | 1.27E-19 | *ME1, TM7SF2, SC5D, HSD3B1, SORD, HMGCR, PGD, PRDX2, FDFT1, GLDC, FAR2, PECR, AKR1A1, PIR, CPOX, DHCR7, GPX3, FASN, SRD5A1, NSDHL, GLRX, DHCR24, ACADM, MSMO1, FADS1, FA2H, BCKDHB, DHRS11, MSRB1, FADS2, DHRS9, HGD, ALDH3B2, CYB5A, SOD2, RDH11, CYP4F8, G6PD, DHFR, ALOX15B, HSD11B1, CYP4F2, MECR, DCXR* |
| BP | GO:0006695 | cholesterol biosynthetic process | 19 | 3.82E-24 | *TM7SF2, ACAA2, EBP, MSMO1, MVD, HMGCR, HMGCS1, FDPS, ACLY, PMVK, FDFT1, G6PD, DHCR7, INSIG1, GGPS1, MVK, IDI1, NSDHL, DHCR24* |
| BP | GO:0006629 | lipid metabolic process | 19 | 5.23E-12 | *SC5D, LDLR, FADS1, ABHD5, PPARG, APOC1, HMGCS1, ALDH3B2, FADS2, CIDEA, ACLY, ACAT2, FAR2, G6PD, CD36, ALOX15B, ACAD8, AACS, PC* |
| BP | GO:0008152 | metabolic process | 18 | 1.54E-10 | *ACAA2, BCAT2, ACO1, BCKDHB, NPL, ECHDC1, TKT, ISOC1, ACSBG1, ACSM3, DBT, ACSL1, INSIG1, UGT2A2, FASN, ACSL3, AGPAT3, ACAA1* |
| BP | GO:0006633 | fatty acid biosynthetic process | 14 | 1.68E-13 | *SC5D, MSMO1, OLAH, FA2H, ACACA, ACLY, ACSM3, ELOVL1, PECR, ELOVL4, FASN, PCCB, MECR, ACSL3* |
| BP | GO:0042493 | response to drug | 12 | 0.003231 | *ACSL1, SORD, PLIN2, PPARG, HMGCS1, GNPAT, SRD5A1, FOSB, AACS, HADH, GAL, SOD2* |
| BP | GO:0035338 | long-chain fatty-acyl-CoA biosynthetic process | 11 | 1.92E-10 | *ELOVL1, ACSL1, ELOVL5, ELOVL4, ACACA, FASN, ACOT1, ACLY, ACSL3, ACSBG1, ACSL5* |
| BP | GO:0006635 | fatty acid beta-oxidation | 10 | 6.78E-09 | *ACOX2, ACAA2, CPT2, ACADM, HIBCH, ACAT2, HADH, SLC27A2, ACAA1, SLC25A17* |
| BP | GO:0008299 | isoprenoid biosynthetic process | 8 | 2.61E-10 | *MVD, HMGCR, FDPS, HMGCS1, GGPS1, MVK, IDI1, FDFT1* |
| BP | GO:0001676 | long-chain fatty acid metabolic process | 7 | 2.83E-07 | *ACSL1, ACOT1, CYP4F2, ACSL3, SLC27A2, ACSBG1, ACSL5* |
| BP | GO:0006631 | fatty acid metabolic process | 7 | 7.44E-05 | *MSMO1, FA2H, ABHD5, FASN, AACS, HADH, MECR* |
| BP | GO:0051289 | protein homotetramerization | 7 | 1.67E-04 | *CTH, GPX3, ACACA, FBP1, CDA, DCXR, SOD2* |
| BP | GO:0007584 | response to nutrient | 7 | 5.27E-04 | *ACSL1, HMGCR, PKLR, PPARG, GNPAT, AACS, ACSL3* |
| BP | GO:0006979 | response to oxidative stress | 7 | 0.004059 | *SGK2, GPX3, ETFDH, MSRB1, IDH1, PRDX2, DHCR24* |
| BP | GO:0036109 | alpha-linolenic acid metabolic process | 6 | 5.43E-07 | *ELOVL1, ACSL1, ELOVL5, FADS1, FADS2, ACAA1* |
| BP | GO:0043651 | linoleic acid metabolic process | 6 | 2.50E-06 | *ELOVL1, ACSL1, ELOVL5, ALOX15B, FADS1, FADS2* |
| BP | GO:0055088 | lipid homeostasis | 6 | 1.82E-04 | *ACOX2, ACADM, PPARG, ACACA, ACAD8, PNPLA3* |
| BP | GO:0030148 | sphingolipid biosynthetic process | 6 | 4.89E-04 | *ELOVL1, ELOVL5, ELOVL4, FA2H, ALDH3B2, CERS4* |
| BP | GO:0006006 | glucose metabolic process | 6 | 0.002236 | *G6PD, SORD, AKR1A1, OAS1, PDHA1, DCXR* |
| BP | GO:0016311 | dephosphorylation | 6 | 0.006544 | *DUSP4, ACP6, FBP1, INPP4B, LPIN1, PON3* |
| BP | GO:0016310 | phosphorylation | 6 | 0.01214 | *CKMT1A, MVK, NADK, GK, PMVK, CDK5* |
| BP | GO:0006636 | unsaturated fatty acid biosynthetic process | 5 | 4.16E-05 | *ELOVL1, ELOVL5, ELOVL4, FADS1, FADS2* |
| BP | GO:0009083 | branched-chain amino acid catabolic process | 5 | 1.13E-04 | *DBT, BCAT2, BCKDHB, ACAD8, HIBCH* |
| BP | GO:0019432 | triglyceride biosynthetic process | 5 | 4.04E-04 | *GK, PNPLA3, LPIN1, AGPAT3, AGPAT1* |
| BP | GO:0009058 | biosynthetic process | 5 | 7.10E-04 | *ALAS1, OLAH, FASN, APMAP, FDFT1* |
| BP | GO:0006641 | triglyceride metabolic process | 5 | 0.001286 | *ACSL1, INSIG1, APOC1, GK3P, GK* |
| BP | GO:0014070 | response to organic cyclic compound | 5 | 0.004484 | *ACSL1, G6PD, PLIN2, FADS1, ACSL3* |
| BP | GO:0042632 | cholesterol homeostasis | 5 | 0.011474 | *SOAT1, ACSM3, TMEM97, NUS1, LDLR* |
| BP | GO:0008203 | cholesterol metabolic process | 5 | 0.0141 | *SOAT1, EBP, LDLR, INSIG1, APOC1* |
| BP | GO:0001889 | liver development | 5 | 0.018705 | *ACADM, HMGCS1, SRD5A1, AACS, SOD2* |
| BP | GO:0032869 | cellular response to insulin stimulus | 5 | 0.021318 | *PKLR, PPARG, SRD5A1, LPIN1, CEACAM1* |
| BP | GO:0001558 | regulation of cell growth | 5 | 0.024144 | *TMEM97, SGK2, TFRC, CLSTN3, CEACAM1* |
| BP | GO:0050830 | defense response to Gram-positive bacterium | 5 | 0.029337 | *HIST1H2BC, CD36, H2BFS, HIST1H2BK, HIST1H2BJ* |
| BP | GO:0033489 | cholesterol biosynthetic process via desmosterol | 4 | 1.00E-05 | *EBP, SC5D, DHCR7, DHCR24* |
| BP | GO:0033490 | cholesterol biosynthetic process via lathosterol | 4 | 1.00E-05 | *EBP, SC5D, DHCR7, DHCR24* |
| BP | GO:0031325 | positive regulation of cellular metabolic process | 4 | 2.49E-05 | *ACACA, FASN, ACLY, AGPAT1* |
| BP | GO:0044539 | long-chain fatty acid import | 4 | 8.52E-05 | *ACSL1, CD36, ACSL3, SLC27A2* |
| BP | GO:0033540 | fatty acid beta-oxidation using acyl-CoA oxidase | 4 | 6.55E-04 | *ACOX2, AMACR, CRAT, ACAA1* |
| BP | GO:0006768 | biotin metabolic process | 4 | 8.26E-04 | *ACACA, HLCS, PCCB, PC* |
| BP | GO:0000038 | very long-chain fatty acid metabolic process | 4 | 8.26E-04 | *ACOT1, CYP4F2, ACAA1, ACSBG1* |
| BP | GO:0008610 | lipid biosynthetic process | 4 | 8.26E-04 | *ACSL1, OLAH, ACLY, FDFT1* |
| BP | GO:0033539 | fatty acid beta-oxidation using acyl-CoA dehydrogenase | 4 | 0.001778 | *ACOX2, ACADM, ETFDH, ACAD8* |
| BP | GO:0002227 | innate immune response in mucosa | 4 | 0.00467 | *HIST1H2BC, H2BFS, HIST1H2BK, HIST1H2BJ* |
| BP | GO:0046487 | glyoxylate metabolic process | 4 | 0.005226 | *DBT, BCKDHB, PDHA1, GLDC* |
| BP | GO:0006694 | steroid biosynthetic process | 4 | 0.009388 | *HSD3B1, SRD5A1, NSDHL, FDFT1* |
| BP | GO:0006654 | phosphatidic acid biosynthetic process | 4 | 0.012022 | *ABHD5, GNPAT, AGPAT3, AGPAT1* |
| BP | GO:0042594 | response to starvation | 4 | 0.012022 | *ACADM, PPARG, GNPAT, AACS* |
| BP | GO:0008654 | phospholipid biosynthetic process | 4 | 0.017266 | *FADS1, ABHD5, AGPAT3, AGPAT1* |
| BP | GO:0019731 | antibacterial humoral response | 4 | 0.022243 | *HIST1H2BC, H2BFS, HIST1H2BK, HIST1H2BJ* |
| BP | GO:0009267 | cellular response to starvation | 4 | 0.026436 | *FADS1, DSC2, SRD5A1, CTSV* |
| BP | GO:0019287 | isopentenyl diphosphate biosynthetic process, mevalonate pathway | 3 | 5.55E-04 | *MVD, MVK, PMVK* |
| BP | GO:0016557 | peroxisome membrane biogenesis | 3 | 0.001101 | *PEX11A, PEX16, PEX3* |
| BP | GO:0019640 | glucuronate catabolic process to xylulose 5-phosphate | 3 | 0.001818 | *SORD, AKR1A1, DCXR* |
| BP | GO:0061732 | mitochondrial acetyl-CoA biosynthetic process from pyruvate | 3 | 0.002702 | *MPC1, MPC2, PDHA1* |
| BP | GO:0019367 | fatty acid elongation, saturated fatty acid | 3 | 0.003749 | *ELOVL1, ELOVL5, ELOVL4* |
| BP | GO:0001561 | fatty acid alpha-oxidation | 3 | 0.003749 | *PEX13, SLC27A2, SLC25A17* |
| BP | GO:0034626 | fatty acid elongation, polyunsaturated fatty acid | 3 | 0.003749 | *ELOVL1, ELOVL5, ELOVL4* |
| BP | GO:0034625 | fatty acid elongation, monounsaturated fatty acid | 3 | 0.003749 | *ELOVL1, ELOVL5, ELOVL4* |
| BP | GO:0033993 | response to lipid | 3 | 0.004953 | *CD36, PPARG, RIDA* |
| BP | GO:0034379 | very-low-density lipoprotein particle assembly | 3 | 0.004953 | *SOAT1, APOC1, ACSL3* |
| BP | GO:0016126 | sterol biosynthetic process | 3 | 0.007818 | *TM7SF2, MSMO1, PMVK* |
| BP | GO:0006098 | pentose-phosphate shunt | 3 | 0.00947 | *G6PD, PGD, TKT* |
| BP | GO:0015908 | fatty acid transport | 3 | 0.011262 | *ACSL1, SLC27A2, SLC25A17* |
| BP | GO:0045717 | negative regulation of fatty acid biosynthetic process | 3 | 0.011262 | *INSIG1, APOC1, CEACAM1* |
| BP | GO:0042761 | very long-chain fatty acid biosynthetic process | 3 | 0.013191 | *ELOVL1, ELOVL5, ELOVL4* |
| BP | GO:0050995 | negative regulation of lipid catabolic process | 3 | 0.019755 | *APOC1, IL1B, CIDEA* |
| BP | GO:0007031 | peroxisome organization | 3 | 0.024741 | *PEX11A, PEX16, PEX3* |
| BP | GO:0006699 | bile acid biosynthetic process | 3 | 0.033061 | *ACOX2, AMACR, SLC27A2* |
| BP | GO:0035902 | response to immobilization stress | 3 | 0.033061 | *PPARG, GAL, SOD2* |
| BP | GO:0006090 | pyruvate metabolic process | 3 | 0.036045 | *ME1, PDHA1, PC* |
| BP | GO:0090314 | positive regulation of protein targeting to membrane | 3 | 0.042308 | *CHP1, PDZK1, CDK5* |
| BP | GO:0019915 | lipid storage | 3 | 0.042308 | *CD36, PLIN2, CIDEA* |
| BP | GO:0046686 | response to cadmium ion | 3 | 0.045581 | *SORD, CYB5A, SOD2* |
| BP | GO:0019369 | arachidonic acid metabolic process | 3 | 0.045581 | *ALOX15B, FADS1, CYP4F2* |
| BP | GO:0045337 | farnesyl diphosphate biosynthetic process | 2 | 0.027207 | *FDPS, GGPS1* |
| BP | GO:1902361 | mitochondrial pyruvate transmembrane transport | 2 | 0.027207 | *MPC1, MPC2* |
| BP | GO:0033384 | geranyl diphosphate biosynthetic process | 2 | 0.027207 | *FDPS, GGPS1* |
| BP | GO:0009051 | pentose-phosphate shunt, oxidative branch | 2 | 0.027207 | *G6PD, PGD* |
| BP | GO:0019322 | pentose biosynthetic process | 2 | 0.027207 | *G6PD, PGD* |
| BP | GO:0019254 | carnitine metabolic process, CoA-linked | 2 | 0.040533 | *ACADM, CRAT* |
| BP | GO:0046167 | glycerol-3-phosphate biosynthetic process | 2 | 0.040533 | *GK3P, GK* |
| BP | GO:0006850 | mitochondrial pyruvate transport | 2 | 0.040533 | *MPC1, MPC2* |
| BP | GO:0006741 | NADP biosynthetic process | 2 | 0.040533 | *ME1, NADK* |
| BP | GO:0034201 | response to oleic acid | 2 | 0.040533 | *ACSL1, AACS* |
| BP | GO:0006740 | NADPH regeneration | 2 | 0.040533 | *G6PD, IDH1* |
| CC | GO:0016021 | integral component of membrane | 82 | 0.03077 | *TM7SF2, IER3, KCNJ15, SC5D, LDLR, SLC15A1, HMGCR, CLSTN3, ECHDC1, TSPAN6, SLC7A5, FDFT1, ELOVL1, FAR2, ADTRP, ELOVL5, PGRMC1, CPOX, PXMP4, INSIG1, MC5R, PXMP2, SRD5A1, AGPAT3, CEACAM1, AGPAT1, DHCR24, FA2H, UBIAD1, CYB5A, PNPLA3, CD36, NUS1, TMPRSS11E, HSD11B1, INPP4B, SLC30A10, SNX13, SLC27A2, HSD3B1, TMEM254, CERS4, SEC14L4, PAQR3, ACSL1, TMED5, DHCR7, UGT2A2, ETFDH, BCAP29, APMAP, MFAP3L, SLC31A1, ACSL3, ACSL5, BEAN1, NSDHL, SOAT1, MUC1, EBP, TMEM97, MSMO1, GABRA4, FADS1, PMEL, FDPS, MPC1, FADS2, MPC2, HILPDA, ADGRG2, RDH11, CYP4F8, TFRC, TMEM164, SLC50A1, SPCS3, DSC2, CYP4F2, SLC46A3, SLC25A16, SLC25A17* |
| CC | GO:0070062 | extracellular exosome | 72 | 1.21E-08 | *CLSTN3, PGD, ECHDC1, TSPAN6, PRDX2, PMVK, SLC7A5, CTTN, SAA2, PGRMC1, GPX3, IL1B, HIST3H2A, GNG4, DNAJC3, CEACAM1, RAB27A, DDC, ACAA2, ACADM, ACO1, FBP1, CHP1, CYB5A, CTSV, CTH, G6PD, ALOX15B, HAO2, PKLR, MVK, WASL, CUX2, SLC27A2, SORD, FKBP5, HIST1H2AE, APOC1, ACAT2, KRT9, AKR1A1, CALML3, FASN, IDH1, DOPEY2, APMAP, GLRX, MUC1, HIST1H2BC, ACY1, SLCO4C1, UPB1, ACACA, CHI3L1, HGD, TKT, ACLY, ISOC1, ADGRG2, SOD2, TST, CKMT1A, TFRC, DSC2, HIST1H2AH, HIBCH, GK, RIDA, SLC46A3, PDZK1, DCXR, PON3* |
| CC | GO:0005829 | cytosol | 68 | 9.80E-05 | *MOCOS, IER3, PPARG, PGD, ECHDC1, ACOT1, PRDX2, HLCS, PEX3, PMVK, SLC7A5, GLDC, IL1B, DNAJC3, SAR1B, DHCR24, DDC, SGK2, PIK3C2G, ACO1, PCTP, FBP1, CHP1, LPIN1, CDK5, CTH, G6PD, MAD2L1, ALOX15B, PKLR, INPP4B, MVK, WASL, PCCB, ME1, SORD, MVD, ABHD5, HMGCS1, OAS1, ZBTB16, HMMR, ACSBG1, PLIN2, AKR1A1, GGPS1, FASN, CDA, IDH1, DOPEY2, GLRX, ACY1, UPB1, ACACA, NPL, FDPS, HGD, MSRB1, NADK, TKT, ACLY, DHFR, GK, FABP7, RIDA, IDI1, AACS, PC* |
| CC | GO:0005739 | mitochondrion | 51 | 7.60E-12 | *ACOX2, CPT2, HLCS, GLDC, PECR, ALAS1, CPOX, GNPAT, PDHA1, ACAD8, HADH, ACAA2, ACADM, ACO1, HAO2, SLC27A2, MECR, PCCB, ME1, APOOL, BCAT2, ACP6, OAS1, ACAT2, ACSL1, ARG2, FASN, IDH1, ACSL5, GLRX, FADS1, AMACR, BCKDHB, FDPS, ACACA, C21ORF33, CIDEA, MPC1, MPC2, CRAT, SOD2, TST, DBT, CKMT1A, GK3P, GK, HIBCH, RIDA, AGR2, PC, SLC25A17* |
| CC | GO:0016020 | membrane | 49 | 2.27E-04 | *TM7SF2, KCNJ15, SORD, SLC15A1, LDLR, FKBP5, CLSTN3, PEX3, PMVK, SLC7A5, HMMR, KRT9, ELOVL1, ACSL1, ELOVL5, DHCR7, PGRMC1, PEX16, FASN, BCAP29, PXMP2, GNPAT, PEX13, APMAP, DNAJC3, AGPAT3, ACSL3, CEACAM1, PHLDA2, AGPAT1, ACSL5, DHCR24, SOAT1, FADS1, UBIAD1, FADS2, CYB5A, ACLY, CDK5, PNPLA3, PPIF, SLC26A3, G6PD, CD36, TFRC, ALOX15B, HSD11B1, ACAA1, SLC25A17* |
| CC | GO:0005789 | endoplasmic reticulum membrane | 46 | 1.11E-15 | *TM7SF2, SC5D, HSD3B1, CLSTN3, FKBP5, HMGCR, CERS4, FDFT1, ELOVL1, FAR2, ACSL1, TMED5, ELOVL5, DHCR7, PGRMC1, INSIG1, PEX16, BCAP29, SRD5A1, SAR1B, AGPAT3, ACSL3, AGPAT1, ACSL5, DHCR24, NSDHL, SOAT1, EBP, MSMO1, FADS1, FA2H, PMEL, UBIAD1, DHRS9, FADS2, CYB5A, PNPLA3, LPIN1, CYP4F8, RDH11, NUS1, MCFD2, HSD11B1, SPCS3, CYP4F2, SLC27A2* |
| CC | GO:0005783 | endoplasmic reticulum | 37 | 2.06E-10 | *TM7SF2, HMGCR, APOC1, OAS1, CERS4, PEX3, ACSBG1, FDFT1, ELOVL1, PLIN2, ELOVL5, ELOVL4, DHCR7, PGRMC1, INSIG1, PEX16, BCAP29, APMAP, SAR1B, DNAJC3, AGPAT3, ACSL3, AGPAT1, ACSL5, DHCR24, NSDHL, SOAT1, EBP, MSMO1, ACO1, FA2H, UBIAD1, CHI3L1, CHP1, CRAT, SPCS3, AGR2* |
| CC | GO:0005777 | peroxisome | 23 | 9.55E-21 | *ACOX2, ACADM, MVD, AMACR, TKT, CRAT, ISOC1, PEX3, PMVK, PEX11A, FAR2, PECR, PXMP4, HAO2, PEX16, GNPAT, IDH1, PEX13, MVK, RIDA, IDI1, ACAA1, SLC25A17* |
| CC | GO:0043231 | intracellular membrane-bounded organelle | 23 | 4.39E-06 | *ACOX2, SC5D, FADS1, ABHD5, PPARG, CYB5A, PEX3, SLC7A5, PECR, CTTN, G6PD, TFRC, PLIN2, DHCR7, NOP16, UGT2A2, PEX13, PDHA1, CYP4F2, PON3, ACAA1, NSDHL, SLC25A17* |
| CC | GO:0005759 | mitochondrial matrix | 18 | 1.51E-06 | *ACADM, BCAT2, BCKDHB, SOD2, GLDC, ACSM3, PPIF, TST, DBT, ALAS1, ARG2, ETFDH, ACAD8, HIBCH, PDHA1, HADH, PCCB, PC* |
| CC | GO:0005743 | mitochondrial inner membrane | 16 | 7.19E-04 | *ACAA2, CPT2, HSD3B1, MPC1, MPC2, CRAT, SOD2, PPIF, TST, CKMT1A, CPOX, ETFDH, SLC25A16, HADH, ACSL5, SLC25A17* |
| CC | GO:0005778 | peroxisomal membrane | 14 | 1.60E-13 | *HMGCR, PEX3, PEX11A, PECR, FAR2, ACSL1, PEX16, PXMP4, PXMP2, GNPAT, PEX13, ACSL3, SLC27A2, SLC25A17* |
| CC | GO:0016324 | apical plasma membrane | 10 | 0.014536 | *MUC1, SLC26A3, CD36, AKR1A1, CYP4F2, ADGRG2, SLC7A5, PDZK1, CEACAM1, RAB27A* |
| CC | GO:0005782 | peroxisomal matrix | 8 | 2.06E-06 | *ACOX2, FAR2, AMACR, HAO2, GNPAT, IDH1, CRAT, ACAA1* |
| CC | GO:0005811 | lipid particle | 8 | 2.40E-05 | *PLIN2, ABHD5, CIDEA, ALDH3B2, HILPDA, PNPLA3, ACSL3, NSDHL* |
|  | GO:0031090 | organelle membrane | 8 | 1.43E-04 | *TM7SF2, CYP4F8, FA2H, PGRMC1, SPCS3, DHRS9, SRD5A1, CYP4F2* |
| CC | GO:0030176 | integral component of endoplasmic reticulum membrane | 8 | 4.30E-04 | *TM7SF2, ELOVL1, FAM134B, ELOVL5, ELOVL4, DHCR7, DHRS9, SLC27A2* |
| CC | GO:0005779 | integral component of peroxisomal membrane | 7 | 2.11E-08 | *PEX11A, PEX16, PXMP2, PEX13, PEX3, SLC27A2, SLC25A17* |
| CC | GO:0005741 | mitochondrial outer membrane | 7 | 0.013627 | *ACSL1, CYB5A, GK3P, GK, LPIN1, ACSL3, ACSL5* |
| CC | GO:0000786 | nucleosome | 6 | 0.0078 | *HIST1H2BC, HIST1H2BK, HIST1H1C, HIST1H2AE, HIST1H2BJ, HIST1H2AH* |
| CC | GO:0043209 | myelin sheath | 6 | 0.049395 | *SYN2, SRD5A1, TKT, PDHA1, TAGLN3, SOD2* |
| CC | GO:0000788 | nuclear nucleosome | 5 | 0.002556 | *HIST1H2BC, H2BFS, HIST1H2BK, HIST1H2BJ, HIST3H2A* |
| CC | GO:0030141 | secretory granule | 5 | 0.016007 | *IL1B, HILPDA, GAL, CTSV, RAB27A* |
| MF | GO:0042803 | protein homodimerization activity | 20 | 0.005146 | *MVD, HMGCR, E2F8, HMGCS1, CIDEA, HLCS, TKT, ZBTB16, PEX11A, MAD2L1, G6PD, TFRC, CPOX, FASN, IDH1, CDA, RIDA, AGR2, CEACAM1, PON3* |
| MF | GO:0042802 | identical protein binding | 18 | 0.026506 | *ACY1, ACADM, SORD, LDLR, PPARG, FBP1, NPL, HGD, ZBTB16, SOD2, ALAS1, CTH, MAD2L1, G6PD, TFRC, GGPS1, MVK, DCXR* |
| MF | GO:0016491 | oxidoreductase activity | 15 | 6.15E-07 | *SC5D, MSMO1, SORD, FADS1, DHRS11, ALDH3B2, FDFT1, FAR2, AKR1A1, HAO2, FASN, ETFDH, HSD11B1, MECR, DCXR* |
| MF | GO:0003824 | catalytic activity | 13 | 1.02E-05 | *ACSM3, ALAS1, ACSL1, BCAT2, SYN2, BCKDHB, AMACR, ECHDC1, FASN, TKT, ISOC1, ACSL3, ACSBG1* |
| MF | GO:0009055 | electron carrier activity | 9 | 3.02E-05 | *ACOX2, ME1, ACADM, AKR1A1, ETFDH, SRD5A1, ACAD8, GLRX, GLDC* |
| MF | GO:0050661 | NADP binding | 7 | 6.82E-06 | *TM7SF2, ME1, G6PD, DHFR, HMGCR, DHCR7, IDH1* |
| MF | GO:0030170 | pyridoxal phosphate binding | 6 | 0.001037 | *MOCOS, DDC, ALAS1, CTH, BCAT2, GLDC* |
| MF | GO:0102391 | decanoate--CoA ligase activity | 5 | 2.21E-06 | *ACSL1, ACSL3, SLC27A2, ACSBG1, ACSL5* |
| MF | GO:0004467 | long-chain fatty acid-CoA ligase activity | 5 | 2.14E-05 | *ACSL1, ACSL3, SLC27A2, ACSBG1, ACSL5* |
| MF | GO:0016746 | transferase activity, transferring acyl groups | 5 | 0.00187 | *ACAA2, DBT, CPT2, CRAT, AGPAT3* |
| MF | GO:0050660 | flavin adenine dinucleotide binding | 5 | 0.011107 | *ACOX2, ACADM, ETFDH, ACAD8, DHCR24* |
| MF | GO:0016740 | transferase activity | 5 | 0.041684 | *MOCOS, FDPS, OAS1, GLRX, FDFT1* |
| MF | GO:0016831 | carboxy-lyase activity | 4 | 9.94E-04 | *DDC, MVD, BCKDHB, ECHDC1* |
| MF | GO:0070402 | NADPH binding | 4 | 9.94E-04 | *DHFR, HMGCR, FASN, SRD5A1* |
| MF | GO:0016627 | oxidoreductase activity, acting on the CH-CH group of donors | 4 | 0.001729 | *TM7SF2, ACOX2, ACADM, ACAD8* |
| MF | GO:0000062 | fatty-acyl-CoA binding | 4 | 0.007636 | *SOAT1, ACOX2, ACADM, ACAD8* |
| MF | GO:0016829 | lyase activity | 4 | 0.009145 | *MOCOS, NPL, ACLY, GLDC* |
| MF | GO:0030145 | manganese ion binding | 4 | 0.030271 | *ME1, ENDOU, IDI1, SOD2* |
| MF | GO:0050833 | pyruvate transmembrane transporter activity | 3 | 5.45E-04 | *SLC16A7, MPC1, MPC2* |
| MF | GO:0016628 | oxidoreductase activity, acting on the CH-CH group of donors, NAD or NADP as acceptor | 3 | 0.002651 | *TM7SF2, DHCR7, DHCR24* |
| MF | GO:0009922 | fatty acid elongase activity | 3 | 0.003679 | *ELOVL1, ELOVL5, ELOVL4* |
| MF | GO:0102338 | 3-oxo-lignoceronyl-CoA synthase activity | 3 | 0.003679 | *ELOVL1, ELOVL5, ELOVL4* |
| MF | GO:0102337 | 3-oxo-cerotoyl-CoA synthase activity | 3 | 0.003679 | *ELOVL1, ELOVL5, ELOVL4* |
| MF | GO:0102336 | 3-oxo-arachidoyl-CoA synthase activity | 3 | 0.003679 | *ELOVL1, ELOVL5, ELOVL4* |
| MF | GO:0052890 | oxidoreductase activity, acting on the CH-CH group of donors, with a flavin as acceptor | 3 | 0.017127 | *ACOX2, ACADM, ACAD8* |
| MF | GO:0003995 | acyl-CoA dehydrogenase activity | 3 | 0.019401 | *ACOX2, ACADM, ACAD8* |
| MF | GO:0003841 | 1-acylglycerol-3-phosphate O-acyltransferase activity | 3 | 0.026921 | *ABHD5, AGPAT3, AGPAT1* |
| MF | GO:0004320 | oleoyl-[acyl-carrier-protein] hydrolase activity | 2 | 0.026948 | *OLAH, FASN* |
| MF | GO:0016296 | palmitoyl-[acyl-carrier-protein] hydrolase activity | 2 | 0.026948 | *OLAH, FASN* |
| MF | GO:0016295 | myristoyl-[acyl-carrier-protein] hydrolase activity | 2 | 0.026948 | *OLAH, FASN* |
| MF | GO:0000248 | C-5 sterol desaturase activity | 2 | 0.026948 | *SC5D, FADS1* |
| MF | GO:0004337 | geranyltranstransferase activity | 2 | 0.026948 | *FDPS, GGPS1* |
| MF | GO:0004769 | steroid delta-isomerase activity | 2 | 0.040149 | *EBP, HSD3B1* |
| MF | GO:0004659 | prenyltransferase activity | 2 | 0.040149 | *NUS1, UBIAD1* |
| MF | GO:0019166 | trans-2-enoyl-CoA reductase (NADPH) activity | 2 | 0.040149 | *PECR, MECR* |
| MF | GO:0003989 | acetyl-CoA carboxylase activity | 2 | 0.040149 | *ACACA, PCCB* |
| MF | GO:0004161 | dimethylallyltranstransferase activity | 2 | 0.040149 | *FDPS, GGPS1* |
| KEGG | hsa01100 | Metabolic pathways | 87 | 1.93E-27 | *TM7SF2, ACOX2, SC5D, HMGCR, PGD, ACOT1, HLCS, PMVK, FDFT1, GLDC, ALAS1, CPOX, ACAD8, PDHA1, HADH, AGPAT3, AGPAT1, DHCR24, ACAA2, DDC, PIK3C2G, ACADM, ACO1, FBP1, ALDH3B2, DHRS9, LPIN1, PNPLA3, CTH, G6PD, ALOX15B, HAO2, PKLR, PLA2G7, HSD11B1, MVK, INPP4B, PCCB, MECR, ACAA1, ME1, BCAT2, SORD, HSD3B1, MVD, OLAH, HMGCS1, CERS4, ACAT2, ACSBG1, ACSL1, AKR1A1, DHCR7, ARG2, GGPS1, FASN, UGT2A2, CDA, IDH1, ACSL3, ACSL5, NSDHL, EBP, ACY1, MSMO1, UPB1, BCKDHB, AMACR, ACACA, FDPS, HGD, NADK, TKT, ACLY, ACSM3, TST, RDH11, CYP4F8, DBT, CKMT1A, DHFR, HIBCH, CYP4F2, GK, IDI1, DCXR, PC* |
| KEGG | hsa01130 | Biosynthesis of antibiotics | 37 | 1.16E-22 | *TM7SF2, SC5D, BCAT2, MVD, HMGCR, PGD, HMGCS1, ACAT2, FDFT1, GLDC, AKR1A1, ARG2, GGPS1, IDH1, PDHA1, HADH, NSDHL, ACAA2, MSMO1, ACY1, ACADM, ACO1, BCKDHB, FDPS, FBP1, ACLY, TKT, DBT, CTH, G6PD, PKLR, HAO2, PLA2G7, MVK, IDI1, PCCB, ACAA1* |
| KEGG | hsa04146 | Peroxisome | 23 | 1.95E-18 | *ACOX2, AMACR, PEX3, CRAT, PMVK, SOD2, PEX11A, FAR2, PECR, ACSL1, PXMP4, HAO2, PEX16, GNPAT, IDH1, PXMP2, PEX13, MVK, ACSL3, SLC27A2, ACAA1, ACSL5, SLC25A17* |
| KEGG | hsa01212 | Fatty acid metabolism | 17 | 2.02E-15 | *ACAA2, ACADM, CPT2, FADS1, ACACA, FADS2, ACAT2, ACSBG1, PECR, ACSL1, ELOVL5, FASN, HADH, MECR, ACSL3, ACAA1, ACSL5* |
| KEGG | hsa01200 | Carbon metabolism | 16 | 2.25E-08 | *ME1, ACADM, ACO1, PGD, FBP1, TKT, ACAT2, GLDC, G6PD, HAO2, PKLR, IDH1, HIBCH, PDHA1, PCCB, PC* |
| KEGG | hsa03320 | PPAR signaling pathway | 14 | 1.76E-09 | *ACOX2, ACADM, CPT2, PPARG, FADS2, ACSBG1, CD36, ACSL1, GK, FABP7, SLC27A2, ACSL3, ACAA1, ACSL5* |
| KEGG | hsa00280 | Valine, leucine and isoleucine degradation | 13 | 2.54E-10 | *ACAA2, ACADM, BCAT2, BCKDHB, HMGCS1, ACAT2, DBT, HIBCH, ACAD8, HADH, AACS, PCCB, ACAA1* |
| KEGG | hsa00900 | Terpenoid backbone biosynthesis | 10 | 4.09E-10 | *NUS1, MVD, HMGCR, FDPS, HMGCS1, GGPS1, MVK, PMVK, ACAT2, IDI1* |
| KEGG | hsa00071 | Fatty acid degradation | 10 | 2.51E-07 | *ACAA2, ACSL1, CPT2, ACADM, ACAT2, HADH, ACSL3, ACAA1, ACSBG1, ACSL5* |
| KEGG | hsa05034 | Alcoholism | 10 | 0.016891 | *DDC, HIST1H2BC, HIST1H2BK, CALML3, HIST1H2AE, HIST1H2BJ, HIST1H2AH, HIST3H2A, FOSB, GNG4* |
| KEGG | hsa00100 | Steroid biosynthesis | 9 | 5.01E-09 | *TM7SF2, SOAT1, EBP, SC5D, MSMO1, DHCR7, NSDHL, FDFT1, DHCR24* |
| KEGG | hsa01230 | Biosynthesis of amino acids | 9 | 1.79E-04 | *CTH, BCAT2, ACY1, ACO1, ARG2, PKLR, IDH1, TKT, PC* |
| KEGG | hsa00061 | Fatty acid biosynthesis | 7 | 1.66E-07 | *ACSL1, OLAH, ACACA, FASN, ACSL3, ACSBG1, ACSL5* |
| KEGG | hsa00062 | Fatty acid elongation | 7 | 1.37E-05 | *ELOVL1, ACAA2, ELOVL5, ELOVL4, ACOT1, HADH, MECR* |
| KEGG | hsa01040 | Biosynthesis of unsaturated fatty acids | 6 | 1.24E-04 | *PECR, ELOVL5, FADS1, ACOT1, FADS2, ACAA1* |
| KEGG | hsa00640 | Propanoate metabolism | 6 | 3.31E-04 | *ACADM, ACACA, ECHDC1, HIBCH, ACAT2, PCCB* |
| KEGG | hsa00620 | Pyruvate metabolism | 6 | 0.00179 | *ME1, PKLR, ACACA, PDHA1, ACAT2, PC* |
| KEGG | hsa00561 | Glycerolipid metabolism | 6 | 0.009055 | *AKR1A1, GK, PNPLA3, LPIN1, AGPAT3, AGPAT1* |
| KEGG | hsa00630 | Glyoxylate and dicarboxylate metabolism | 5 | 0.002771 | *ACO1, HAO2, ACAT2, PCCB, GLDC* |
| KEGG | hsa00650 | Butanoate metabolism | 5 | 0.002771 | *ACSM3, HMGCS1, AACS, ACAT2, HADH* |
| KEGG | hsa00020 | Citrate cycle (TCA cycle) | 5 | 0.00411 | *ACO1, IDH1, ACLY, PDHA1, PC* |
| KEGG | hsa01210 | 2-Oxocarboxylic acid metabolism | 4 | 0.005834 | *BCAT2, ACY1, ACO1, IDH1* |
| KEGG | hsa00030 | Pentose phosphate pathway | 4 | 0.025825 | *G6PD, PGD, FBP1, TKT* |
| KEGG | hsa00410 | beta-Alanine metabolism | 4 | 0.030766 | *ACADM, UPB1, ALDH3B2, HIBCH* |
| KEGG | hsa00040 | Pentose and glucuronate interconversions | 4 | 0.036168 | *SORD, AKR1A1, UGT2A2, DCXR* |

**Notes**: Count, the number of DEGs.

**Abbreviations**: GO, Gene Ontology; KEGG, Kyoto Encyclopedia of Genes and Genomes; DEGs, differentially expressed genes; MF, molecular function; CC, cellular component; BP, biological process.
